# ^11^B NMR Together with Infrared Spectroscopy Provides Insight into Structural Elucidation of Quadrivalent Diazaborines & Cyclic Boronate Esters: Intriguing & Little-Explored

**DOI:** 10.3390/molecules29214998

**Published:** 2024-10-22

**Authors:** Ashley L. Dey

**Affiliations:** Chemistry Research Laboratory, Department of Chemistry, University of Oxford, 12 Mansfield Road, Oxford OX1 3TA, UK; uccaald@ucl.ac.uk

**Keywords:** ^11^B NMR, IR spectroscopy, borazaros, oxazaboron derivatives, *in crystallo*, caged cyclic boronate esters, 2-formylphenylboronic acid (2-FPBA), heterocycles, synthetic and medicinal chemistry

## Abstract

Imidazo-fused diazaborines, which serve as intermediary structures somewhat alongside benzene and borazine, had been of particular interest to Dewar and Snyder more than 60 years ago. To this end, Dewar utilised his ‘π-*complex theory*’so as to represent ‘*borazaros*’as a ‘*quadrivalent*’ species; however, sadly, modern representations have deviated and leapt into ‘*trivalent*’ counterparts. Bonding in boron species has never been straightforward, to such an extent that the orthodox ‘*ethane*’ like diborane, *i.e.*, H_3_B–BH_3_, which conformed to the paradigmatic rules of molecular structure, in particular, hybridisation and electronegativity, was later evolved to a more realistic ‘*3-centre 2-electron*’ bonding so as to give the lie to the purported diborane structures of X-ray diffractors. Herein ^11^B NMR together with IR spectroscopy sheds light on the nature of bonding in borazaros, and ‘*caged*’ cyclic oxazaborons so as to reinforce, and reinvigorate the old literature, which could be of interest to both the synthetic, and medicinal chemist alike.

## 1. Introduction

Unlike ^1^H and ^13^C with a ‘*nuclear spin’ I* = ½, both naturally occurring isotopes of boron, *i.e.*, ^10^B (*I* = 3) and ^11^B (*I* = 5/2), are NMR active; however, the latter is more versatile than the former for NMR investigations by virtue of the fact that ^10^B has a natural abundance of only 19.6%, whereas ^11^B exhibits a natural abundance of 80.4% [1]; as such, ^10^B comes with a much lower receptivity, *i.e.*, receiver gain, and a quadrupole moment almost thrice as much as that for ^11^B, which broadens the ^10^B NMR signals. Moreover, the Larmor frequency of ^10^B is almost three times lower than that for ^11^B, resulting in a much lower signal resolution, and coupling constants, *i.e.*, *J* (in Hz) in ^10^B NMR spectra [2]. Despite these drawbacks, ^10^B NMR can be particularly useful in niche mechanistic studies such as rearrangements [3]. NMR spectroscopy, much to contemporary efforts, has proved exceptionally advantageous in elucidating structures of boron compounds, and their behaviour in solutions [4,5]. The extrapolation of NMR activities of other common NMR active nuclei, such as ^1^H, ^13^C, ^15^N, ^31^P, *etc*., to ^11^B NMR suggests that the following factors should determine chemical shifts (δ) in ^11^B NMR spectra:

- Electron density;
- Coordination number;
- Hybridisation;
- Ring current.

**Figure 1:**
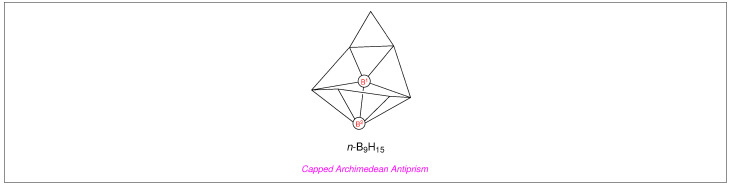
Despite having identical coordination and chemical environments, the signals from B^1^ and B^2^ are the furthest apart from each other in ^11^B NMR spectrum of *n*-B_9_H_15_. Both B^1^ and B^2^ are connected to five other boron atoms and one proton in the capped Archimedean antiprism *n*-B_9_H_15_; *nota bene*: for simplicity, protons are omitted from the borane cluster.

However, the above is found not to be true for many ^11^B NMR spectra. For example, the signals from B^1^ and B^2^ in the ^11^B NMR spectrum of *n*-B_9_H_15_ are ≈64.7 ppm apart, such that B^2^ resonates at the highest field and B^1^ at the lowest field in the aforementioned ^11^B NMR spectrum [6], in spite of both B^1^ and B^2^ in *n*-B_9_H_15_ exhibiting identical connectivity, hybridisation, and even immediate bonding environment (Figure 1).

Similar, but different to the ^11^B NMR spectrum of *n*-B_9_H_15_ Fan *et al.* [7] have very recently discovered that the ^11^B{^1^H} NMR signals from the boron atoms in B_3_H_7_ coordinated 4-methylene dihydropyridine and ethyl 4-isopropyl 3-carboxylate dihydropyridine are reversed compared to that of their parent complexes: the top boron atom connected to the pyridinyl nitrogen is downfielded, and the two base boron atoms resonate at a much higher field in the aforementioned ^11^B{^1^H} NMR spectra, in marked contrast to the general trend observed for most B_3_H_7_ adducts [7].

On the other hand, in stark contrast to other NMR active nuclei, electron density does not appear to preponderate over other factors in determining the position of chemical shifts in ^11^B NMR spectra, as the boron atoms of higher electron density in B_7_$H_{7}^{2-}$ are less shielded than their counterparts of lower electron density in the same cluster [8]. Intriguingly, Ksenofontov *et al.* [9] have attempted to devise an accurate machine learning method for predicting the ^11^B NMR chemical shifts of BODIPYs; however, it has been borne out that the peripherally halogenated BODIPYs are incongruent to an extent that nearly 41% of the aforementioned compounds in their dataset were outliers, and that this should be so is a corollary of, *inter alia*, caveats of the model for not factoring in the effects of spin–orbit interactions. To this end, there are no hard and fast rules as yet to fully predict or explain some of the anomalies that have been observed for the ^11^B NMR spectra, and each and every single boron compound can give rise to distinctive and unexpected ^11^B NMR spectra, which have been observed for the novel boronate esters herein and form the basis of the discussions below.

## 2. Results and Discussion

**Figure 2:**
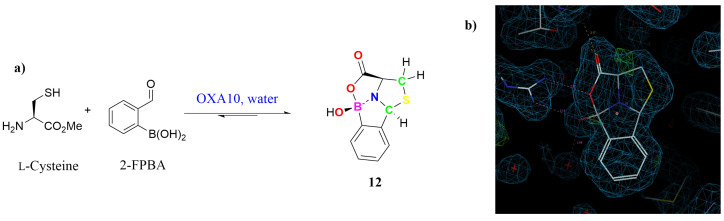
(**a**) *in crystallo* Dynamic combinatorial chemistry of L-cysteine methyl ester with 2-FPBA on the surface of OXA-10; (**b**) X-ray crystal structure of the boronate ester **12** on OXA-10 (*courtesy of Dr Jürgen Brem*).

**Figure 3:**
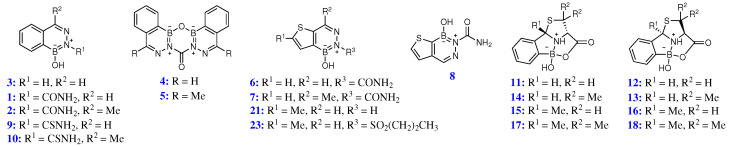
Benzodiazaborines, diazaborines and boronate esters.

Recent protein-directed dynamic combinatorial chemistry studies of L-cysteine derivatives with 2-formylphenylboronic acid (2-FPBA) on the surface, *i.e.*, *in crystallo* of oxacillinases, *e.g.*, OXA-10 (unpublished), brought home an intriguing quadrivalent cyclic boronate ester, *i.e.*, **12** (Figure 2), given that the same observed ‘*tetracyclic caged*’ structure for **12** has been reported in solution elsewhere [10], it is conceivable that based on the observed spectroscopic and X-ray crystallographic data, boronate esters **11** and **13**–**18** (Figure 3) should also follow suit.

Inasmuch as both the diazaborines and boronate esters herein presumably almost certainly feature ‘*quadrivalent*’ boron species, a comparison of chemical shifts (*vide infra*) in ^11^B NMR spectra of the thus obtained diazaborines and boronate esters (Figure 3) not only provides structural insight into these intriguing scaffolds, but also paves the way for a better understanding of bonding in such boron species, and salient factors in their ^11^B NMR resonance frequencies.

Reference has already been made to the unpredictability and irregularity of trends in ^11^B NMR resonance frequencies (*vide supra*); however, typically, though it need not of necessity, the vacant 2*p_z_* orbital of boron plays a crucial role in the position of chemical shifts in ^11^B NMR spectra. Strictly speaking, trivalent boron species resonate at much lower fields than their quadrivalent counterparts. For instance, trimethyl borane as a true trivalent boron species resonates at the lowest field, whereas trimethyl borate as a somewhat quadrivalent boron species with considerable *π*-bonding character resonates at much higher fields in ^11^B NMR. Equally, quite recently Li *et al.* [11] have also observed that increasing the number of SP^3^ hybridised boron atoms in aqueous solutions of borax shifts the corresponding chemical shifts to a higher field in their observed ^11^B NMR spectra. The belief that the determinant factor in the position of chemical shifts in ^11^B NMR spectra is the *π*-donating ability of substituents on boron, implies that diethylamino substituents on boron should bring about upfield shifts for the ^11^B signals in their NMR spectra, relative to that from –OMe by virtue of the fact that –NEt_2_ is mesmerically more *π*-donating than –OMe. However, this effect appears to be reversed in aliphatic boron species, as such B(NEt_2_)_3_ resonates at a much lower field in ^11^B NMR than that observed for B(OMe)_3_ [12].

The chemical shift $\delta_{R}^{C}$ (in ppm) between a compound *C* and reference *R* is given by the following equation:

(1) $$\delta_{R}^{C}=10^{6}\left(H_{C}-H_{R}\right)/H_{R}\approx 10^{6}\left({℘}_{C}-{℘}_{R}\right)$$

where *H_C_* and *H_R_* are the resonance frequencies of the applied magnetic field for compounds *C* and *R,* respectively, and ${℘}_{C}$ and ${℘}_{R}$ are the resulting shielding tensors. Ramsey [13,14], devised an expression for calculating the NMR shielding tensor *℘*; as such, for most solution NMR applications, the shielding tensor *℘* can be replaced by a scalar quantity σ to give the following equation:

(2) $$\sigma =\frac{e^{2}}{2mc^{2}}\langle 0|\sum_{j}\left(x_{j}^{2}+y_{j}^{2}\right)/r_{j}^{3}|0\rangle$$

Neglecting the deferential overlaps of the above expression can lead to the following simplified expression for the chemical shift $\delta_{i}$ of the nucleus of atom *i* [15]:

(3) $$\delta_{i}=Aq_{i}+\sum_{\left(j\neq i\right)}Bq_{j}/r_{ij}^{3}$$

where *q_i_* is the π-electron density of atom *i, r_ij_* is the internuclear distance between atom *i* and *j,* and *A* and *B* are constants. To this end, another salient factor other than the π-donating capabilities of the substituents attached to boron in ^11^B NMR would be the ‘*electronic cloud*’ of the substituents attached to boron, *i.e.*, the availability of lone pairs above and below the plane of the π-bond [15]. As exemplified by the last equation above (Equation (3), atoms *j* with lone pairs orthogonal to the plane of the covalent bond would have a significant impact on the chemical shift by virtue of a large *q_j_* and small *r_ij_* (Table 1).

Analysis of the ^1^H, ^13^C and ^11^B NMR data above (Table 1), on looking more closely, reveals that whilst the boron in 2-FPBA has a much greater trivalent character, less shielded and non-aromatic relative to that in quadrivalent diazaborines which should be more shielded, the boron atoms in the diazaborines togehter with that in 2-FPBA all resonate at about the same field in their ^11^B NMR; on the other hand, the proton of the formyl group in 2-FPBA, resonates at a much lower field than that from γ-C*H* of the benzofused-imadazo ring in ^1^H NMR by virtue of greater shielding that the γ-C*H* moieties of benzofused-imadazo rings experience, which is in marked contrast to that observed for ^11^B NMR. The same is also true for ^13^C NMR data, the γ-*C*H and γ-*C*Me of the imidazo rings are significantly upshifted compared to the non-ring carbon of the formyl group in 2-FPBA (Table 1). Intriguingly, the solvation effect of methanol-$d_{4}$ on benzodiazaborine **4** (*vide infra*) relative to that of acetonitrile-$d_{3}$ for its closely related benzodiazaborine **5** has clearly been echoed by both the upshifted and downshifted signals from boron and γ-*C*H of benzodiazaborine **4** in its ^11^B and ^13^C NMR spectra, respectively. The ^11^B chemical shifts from the boronate esters, in comparison with those from the diazaborines, exhibit a whopping three-fold upfield shift in their spectra, in spite of the fact that both species are quadrivalent, and potentially negatively charged.

**Figure 4:**
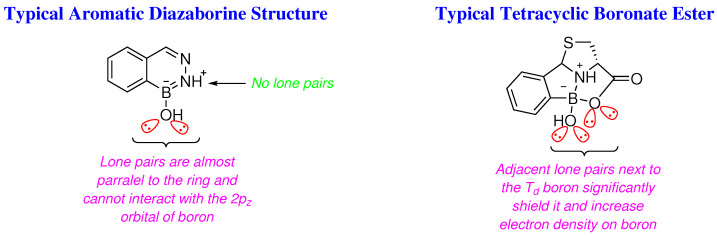
The accessible electronic cloud of the oxygens on boron can account for the upfield shift in ^11^B NMR of the ‘*tetracyclic caged*’ boronate esters.

Whilst the aromaticity of diazaborines can play a crucial role in the observed data, as illustrated above (Equation (3)), the electronic cloud of heteroatoms with available lone pairs adjacent to the boron centre in question can account for the aforementioned large upfield shift of almost 20 ppm in their ^11^B NMR spectra (Figure 4).

**Figure 5:**
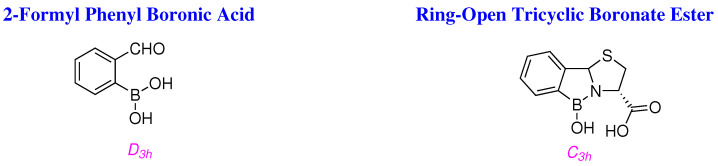
Both ^11^B centres *i.e.*, D_3*h*_ in 2-FPBA, and C_3*h*_ in the ‘*ring-open*’ boronate estersshould resonate with similar frequencies in ^11^B NMR by virtue of similar bonding and shielding effects; however, the boron signal is upshifted in ^11^B NMR spectra of ’*ring-closed*’ boronate esters (Table 1).

In view of the above, it is conceivable that the boronate esters are tetracyclic and ‘*ring-closed*’, as if they were ‘*ring-open*’ and tricyclic, the thus obtianed C_3*h*_ boron species would be less shielded, and would absorb at about the expected field in ^11^B NMR, just like that of 2-FPBA with a similar chemical environment and shielding effects (Figure 5).

**Figure 6:**
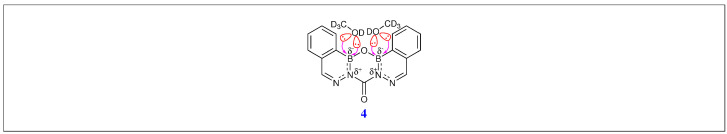
No matter what the hybridisation of boron, the solvation effect of methanol-$d_{4}$ is sufficient to significantly shield the boron centres in benzodiazaborine **4**.

The effects of the electronic cloud of the adjacent substituents on boron (Figure 4) is further evident from the upshifted signal of the boron in benzodiazaborine **4** in its ^11^B NMR spectrum in methanol-$d_{4}$, *i.e.*, $\delta_{B}$ 16.2 (Figure 6), relative to that obtained in acetonitrile-$d_{3}$ for benzodiazaborine **5**, *i.e.*, $\delta_{B}$ 28.3 (Table 1).

**Figure 7:**
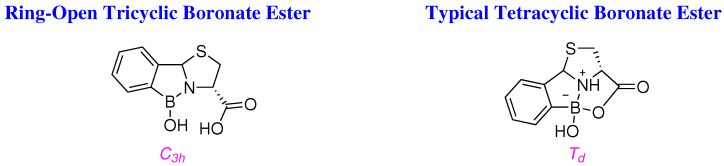
The tricyclic boronic acid intermediate sets the stage for further cyclisation to furnish the isolated tertacyclic caged boronate esters *cf.* (Figure 3), the carbonyl IR frequencies *i.e.*, ≈ 1730–1710 $cm^{-1}$ of which are in agreement with that expected for esters.

Further support for the assertion that the aforementioned boronate esters (Figure 3) are ‘*ring-closed*’ and tetracyclic is provided by the upfield shift of their δ-*C*H and δ-*C*Me signals in ^13^C NMR, relative to that observed for the *C*=O of their precursors *i.e.*, 2-FPBA and 2-acetylphenylboronic acid (2-APBA), which suggests that the D_3*h*_ carbon of the formyl or acyl moieties, is now a $T_{d}$ centre, bonded to four substituents, and fully saturated. This arrangement brings the carboxyl moiety in the ‘*ring-open*’ structure in close proximity to the boronic acid so as to further cyclise, close the ring and form a ‘*tetracyclic caged*’ structure (Figure 7). This is in agreement with the observed IR frequencies *i.e.*, ≈1730–1710 $cm^{-1}$ expected for esters (Table 1), as the parent carboxyl moieties should resonate at lower frequencies *i.e.*, ≈1600–1550 $cm^{-1}$.

## 3. Materials and Methods

### 3.1. General Materials and Instruments

All reagents and solvents in the experiments were of reagent grade quality. They were obtained from commercial sources and used without further purification unless otherwise stated. Diethyl ether, herein, is referred to as ether. Furthermore, 2-FBPA, 2-APBA (*purum* 95.0+%) and sodium phosphate monobasic (*puriss*. anhydrous, >99.0%) were from Sigma-Aldrich^®^ Chemical Co. (Fluka, Germany). Semicarbazide hydrochloride (*puriss.* 99.0%) was from Alfa Aesar^®^ (Guilford, England). The water used was from a Millipede Milli-Q EDM Water Purification A10 unit (Merck, Germany) or was HPLC-grade. High performance liquid chromatography–mass spectrometry (LC-MS) analyses were performed using an Agilent UPLC SQD (USA) instrument equipped with a Chromolith^®^ (ACE, England), C_18_, 2.1 × 50 mm column. The eluent was 0.1% formic acid (v/v) in water (solvent A)/acetonitrile (solvent B) over a 5–95% acetonitrile gradient, with monitoring at 214 nm over 10 min. Preparative reversed phase high performance liquid chromatography (RP-HPLC) was performed using a Shimadzu SPD-20A (Kyoto, Japan) equipped with a Waters Sunfire (USA), C_18_, 250 × 21.2 mm column. The eluent was 0.1% TFA (v/v) in H_2_O (solvent A) and 0.1% TFA (v/v) in MeCN (solvent B) over various MeCN gradients and flow rates as indicated, with monitoring at 214 nm. Electrospray ionisation mass spectrometry was performed using an Agilent single quadrupole unit equipped with CTC-PAL. High-resolution electrospray mass spectra were obtained on a Waters LCT Premier XE spectrometer (USA). The melting points were determined using a Gallenkamp MFG 595 010 melting point apparatus (Synoptics Ltd., Cambridge, England) and a Reichert Austria hot stage microscope (Vienna, Austria). ^1^H, ^13^C and ^11^B NMR spectra were recorded on a Bruker AVIII HD 400 unit or a Bruker AVIIIHD 600 (Nasdaq, USA) equipped with prodigy N_2_ broadband CryoProbe unit, as well as a Bruker AVIII 700 unit (Nasdaq, USA) equipped with TCI H/C/N helium CryoProbe operating (Nasdaq, USA) at 700, 600, 400 MHz, 193, 151, 101 MHz, 225, 193 and 128 MHz, respectively, using CDCl_3_, D_2_O, methanol-$d_{4}$ and acetonitrile-$d_{3}$ as solvents. Chemical shifts (δ) are given in parts per million (ppm) relative to the internal tetramethylsilane (TMS). The coupling constants are given in Hz. The abbreviations s, d, t, td, dd, dq and m correspond to singlet, doublet, triplet, triplet of doublets, doublet of doublets, doublet of quartets, and multiplet, respectively. The IR spectra were recorded on a Bruker Tensor 27 Fourier transform infrared spectrometer (Nasdaq, USA); the abbreviations br, w, m, s and vs correspond to broad, weak, medium, sharp and very sharp, respectively (provisional assignments are given); ${\left[\alpha \right]}_{D}$ values are given in $10^{-1}$ deg cm^2^ g^−1^ at the specified temperature. The reaction products were identified by their NMR, IR spectra and/or otherwise by LC-MS analyses.

### 3.2. General Procedure for Synthesis of Boronic Acid and Ester Heterocycles

The benzodiazaborines, diazaborines and boronate esters were generally synthesised according to the literature procedure [16] with the following modification: the boronic acid (50 mM) and the nucleophile (50 mM) in 0.10 M PBS buffer (pH ≈ 7.4) were nutated for 2.0 min at ambient temperature. If a solid residue was precipitated, the slurry was centrifuged for 20 min at 4.0 °C and washed with water twice, followed by lyophilisation to furnish the desired semi-pure product. In cases where the product did not precipitate, it was purified using preparative RP-HPLC–0.1% TFA (*v*/*v*) in H_2_O (solvent A)/ 0.1% TFA (*v*/*v*) MeCN (solvent B) over a 5–50% MeCN gradient and monitored at 124 nm over 40 min at 40 °C. Samples of the semi-pure products from the aforementioned sedimentation process were also purified using the above preparative RP-HPLC for analytical characterisation purposes.

### 3.3. 1-Hydroxybenzo-[*d*][1,2,3]-diazaborinine-2(1*H*)-carboxamide (***1***)

Benzodiazaborine **1** was synthesised from 2-FPBA and semicarbazide according to the general procedure [16]. The precipitated powder was collected and purified using RP-HPLC (retention time: 12.2–12.5 min) (5.0–50% TFA (0.1% v/v) in MeCN/ TFA (0.1% v/v) in H_2_O over 40 min; 3.0 mL min^−1^, 40 °C) to furnish the desired compound **1** as a white fluffy powder (76 mg; 80%); mp 197–200 °C (*dec.*) (lit. [17] 295–299 °C *dec*.); $\nu_{max}$/$cm^{-1}\;$ (ATR) 3418w (N–H asymmetric), 3140br (N–H symmetric), 2925w (C–H), 1673s (C=O), 1297m (C–N), 1219m (C–B); $\delta_{H}$ (600 MHz, acetonitrile-$d_{3}$) 8.17–8.12 (1 H, m, C*H*-Ar), 8.08 (1 H, s, γ-C*H*), 7.79 (2 H, m, 2 × C*H*-Ar), 7.70 (1 H, td, *J* 7.0 and 1.5, C*H*-Ar); $\delta_{C}$ (151 MHz; acetonitrile-$d_{3}$) 129.0–135.8 (4 × *C*H-Ar and 2 × *C*-Ar), 142.7 (γ-*C*H), 164.1 (*C*=O); $\delta_{B}$ (193 MHz, acetonitrile-$d_{3}$) 29.8; LC-MS (retention time: 5.6–5.9 min) m/z (ESI) 190 (M^+^ + H, 100%); HRMS (ESI) calcd. for C_8_H_8_BN_3_O_2_ [M + H]^+^m/z = 190.0782, found: 190.0784.

### 3.4. 1-Hydroxy-4-methylbenzo[*d*][1,2,3]-diazaborinine-2(1*H*)-carboxamide (***2***)

Benzodiazaborine **2** was synthesised from 2-APBA and semicarbazide according to the general procedure [16]. The precipitated powder was collected and purified using RP-HPLC (retention time: 12.7–13.0 min) (5.0–50% TFA (0.1% v/v) in MeCN/ TFA (0.1% v/v) in H_2_O over 40 min; 3.0 mL min^−1^, 40 °C) to furnish the desired compound **2** as a white fluffy powder (71 mg; 70%); mp 203 °C(*dec.*); $\nu_{max}$/$cm^{-1}\;$ (ATR) 3389w (N–H asymmetric), 3205br (N–H symmetric), 1687s (C=O), 1311s (C–N), 1155m (C–B); $\delta_{H}$ (600 MHz, acetonitrile-$d_{3}$) 8.15–8.13 (1 H, d *J* 12.0, C*H*-Ar), 7.90–7.89 (1 H, d, *J* 6.0, C*H*-Ar), 7.80–7.77 (1 H, td, *J* 12.0 and 6.0, C*H*-Ar), 7.68–7.66 (1 H, td, *J* 12.0 and 6.0, C*H*-Ar), 2.56 (3 H, s, C$H_{3}$); $\delta_{C}$ (151 MHz; acetonitrile-$d_{3}$) 20.2 (*C*H_3_), 126.7–135.8 (4 × *C*H-Ar and 2 × *C*-Ar), 146.9 (γ-*C*H), 164.0 (*C*=O); $\delta_{B}$ (193 MHz, acetonitrile-$d_{3}$) 29.8; LC-MS (retention time: 6.0–6.4 min) m/z (ESI) 204 (M^+^ + H, 100%); HRMS (ESI) calcd. for C_9_H_10_BN_3_O_2_ [M + H]^+^m/z = 204.0939, found: 204.0941.

### 3.5. Benzo[*d*][1,2,3]-diazaborinin-1(2*H*)-ol (***3***)

Benzodiazaborine **3** was synthesised from 2-FPBA and tert-butyl carbazate according to the general procedure [16]. The clear reaction mixture was lyophilised, and the residue was purified using RP-HPLC (retention time: 9.7–10.1 min) (5.0–50% TFA (0.1% v/v) in MeCN/ TFA (0.1% v/v) in H_2_O over 40 min; 3.0 mL min^−1^, 40 °C) to furnish the desired compound **3** as a white powder (38 mg; 31%); mp 232–239 °C (*dec.*); $\nu_{max}$/$cm^{-1}\;$ (ATR) 3323m (N–H), 1558–1440s (C=C), 1380s (B–O), 1342s (B–N); $\delta_{H}$ (600 MHz, acetonitrile-$d_{3}$) 8.10–8.08 (1 H, d, *J* 8.0, C*H*-Ar), 7.96 (1 H, s, γ-C*H*), 7.71–7.70 (2 H, m, 2 × C*H*-Ar), 7.62–7.58 (1 H, m, C*H*-Ar); $\delta_{C}$ (151 MHz; acetonitrile-$d_{3}$) 127.8–136.4 (4 × *C*H-Ar and 2 × *C*-Ar), 140.4 (γ-*C*H); $\delta_{B}$ (193 MHz, acetonitrile-$d_{3}$) 27.7; LC-MS (retention time: 4.5 min) m/z (ESI) 147 (M^+^ + H, 100%); HRMS (ESI) calcd. for C_7_H_7_BN_2_O [M + H]^+^m/z = 147.0724, found: 147.0721.

### 3.6. 8*H*-Benzo[4,5][1,2,3]diazaborinino[3,2-*b*]benzo[4,5][1,2,3]diazaborinino[2,3-e][1,3,5,2,6]oxadiazadiborinin-8-one (***4***)

Benzodiazaborine **4** was synthesised from 2-FPBA and carbazide according to the general procedure [16]. The clear reaction mixture was lyophilised, and the residue was purified using RP-HPLC (retention time: 10.1–10.5 min) (5.0–50% TFA (0.1% v/v) in MeCN/ TFA (0.1% v/v) in H_2_O over 40 min; 3.0 mL min^−1^, 40 °C) to yield the desired compound **4** as a white powder (11 mg; 7.0%); mp 221–225 °C (*dec.*); $\nu_{max}$/$cm^{-1}\;$ (ATR) 2928w (C–H), 1682s (C=O), 1209m (C–B); $\delta_{H}$ (600 MHz, methanol-$d_{4}$) 8.35 (2 H, s, γ-C*H*), 8.23 (2 H, d, *J* 7.5, 2 × C*H*-Ar), 7.83–7.75 (4 H, m, 4 × C*H*-Ar), 7.70 (2 H, t, *J* 7.5, 2 × C*H*-Ar); $\delta_{C}$ (151 MHz; methanol-$d_{4}$) 130.4–133.8 (8 × *C*H-Ar and 2 × *C*-Ar), 146.8 (γ-*C*H), 175.5 (*C*=O); $\delta_{B}$ (193 MHz, methanol-$d_{4}$) 16.2; LC-MS (retention time: 4.06 min) m/z (ESI) 301 (M^+^ + H, 100%); HRMS (ESI) calcd. for C_15_H_12_B_2_N_4_O_3_ [M + H]^+^m/z = 301.1063, found: 301.1060.

### 3.7. 5,11-Dimethyl-8*H*-benzo[4,5][1,2,3]diazaborinino[3,2-*b*]benzo[4,5][1,2,3]diazaborinino[2,3-e][1,3,5,2,6]oxadiazadiborinin-8-one (***5***)

Benzodiazaborine **5** was synthesised from 2-APBA and carbazide according to the general procedure [16]. The clear reaction mixture was lyophilised, and the residue was purified using RP-HPLC (retention time: 12.2–12.5 min) (5.0–50% TFA (0.1% v/v) in MeCN/ TFA (0.1% v/v) in H_2_O over 40 min; 3.0 mL min^−1^, 40 °C) to furnish the desired compound **5** as a white fluffy powder (3.0 mg; 2.0%); mp 244–258 °C (*dec.*); $\nu_{max}$/$cm^{-1}\;$ (ATR) 3399br (O–H), 2923w ($C_{{sp}^{3}}$–H), 1683s (C=O), 1210s (C–B); $\delta_{H}$ (600 MHz, acetonitrile-$d_{3}$) 8.56–8.54 (2 H, d, *J* 8.0, 2 × C*H*-Ar), 8.09–8.07 (2 H, d, *J* 8.0, 2 × C*H*-Ar), 7.97–7.94 (2 H, dd, *J* 12.0 and 8.0, 2 × C*H*-Ar), 7.88–7.84 (2 H, dd, *J* 12.0 and 8.0, 2 × C*H*-Ar), 2.77 (6 H, s, 2 × C$H_{3}$); $\delta_{B}$ (193 MHz, acetonitrile-$d_{3}$) 28.3; LC-MS (retention time: 7.16 min) m/z (ESI) 329 (M^+^ + H, 100%) 346 (M^+^ + H_3_O, 20); HRMS (ESI) calcd. for C_17_H_14_B_2_N_4_O_2_ [M + H]^+^m/z = 329.1376, found: 329.1373.

### 3.8. 1-Hydroxythieno[3,2-*d*][1,2,3]diazaborinine-2(1*H*)-carboxamide (***6***)

Diazaborine **6** was synthesised from 2-FTBA and semicarbazide according to the general procedure [16]. The clear reaction mixture was lyophilised, and the residue was purified using RP-HPLC (retention time: 17.1–18.2 min) (5.0–50% TFA (0.1% v/v) in MeCN/ TFA (0.1% v/v) in H_2_O over 40 min; 3.0 mL min^−1^, 40 °C) to furnish the desired compound **6** as a pale-yellow powder (60 mg; 61%); mp 273 °C (*dec.*); $\nu_{max}$/$cm^{-1}\;$ (ATR) 3500–3200br (H-bonding O–H), 1698s (C=O), 1425vs (B–O), 1331s (B–N), 1287m (C–B); $\delta_{H}$ (600 MHz, acetonitrile-$d_{3}$) 8.20 (1 H, s, γ-C*H*), 7.80–7.79 (1 H, d, *J* 5.0, C*H*-Ar), 7.58–7.57 (1 H, d, *J* 5.0, C*H*-Ar); $\delta_{C}$ (151 MHz; acetonitrile-$d_{3}$) 127.3–135.3 (2 × *C*H-Ar and 2 × *C*-Ar), 140.0 (γ-*C*H), 164.0 (*C*=O); $\delta_{B}$ (193 MHz, acetonitrile-$d_{3}$) 28.6; LC-MS (retention time: 5.8 min) m/z (ESI) 196 (M^+^ + H, 100%), 373 (2 × M^+^ + H, 6.0); HRMS (ESI) calcd. for C_6_H_6_BN_3_O_2_S [M + H]^+^m/z = 196.0347, found: 196.0346.

### 3.9. 1-Hydroxy-4-methylthieno[3,2-*d*][1,2,3]diazaborinine-2(1*H*)-carboxamide (***7***)

Diazaborine **7** was synthesised from 2-ATBA and semicarbazide according to the general procedure [16]. The clear reaction mixture was lyophilised, and the residue was purified using RP-HPLC (retention time: 18.3–19.0 min) (5.0–50% TFA (0.1% v/v) in MeCN/ TFA (0.1% v/v) in H_2_O over 40 min; 3.0 mL min^−1^, 40 °C) to furnish the desired compound **7** as a white fluffy powder (75 mg; 72%); mp 278 °C (*dec.*); $\nu_{max}$/$cm^{-1}\;$ (ATR) 3448w (N–H asymmetric), 3261br (N–H symmetric), 1699s (C=O), 1421s (B–O), 1350s (B–N), 1286vs (C–B), 663vs (B–O); $\delta_{H}$ (600 MHz, acetonitrile-$d_{3}$) 7.76–7.75 (1 H, d, *J* 6.0, C*H*-Ar), 7.57–7.56 (1 H, d, *J* 6.0, C*H*-Ar), 2.49 (3 H, s, C$H_{3}$); $\delta_{C}$ (151 MHz; acetonitrile-$d_{3}$) 20.9 (*C*H_3_), 130.3–131.3 (2 × *C*H-Ar and 2 × *C*-Ar), 142.4 (γ-*C* and *C*=O); $\delta_{B}$ (193 MHz, acetonitrile-$d_{3}$) 28.4; LC-MS (retention time: 6.3 min) m/z (ESI) 210 (M^+^ + H, 100%); HRMS (ESI) calcd. for C_7_H_8_BN_3_O_2_S [M + H]^+^m/z = 210.0503, found: 210.0502.

### 3.10. 1-Hydroxythieno[2,3-*d*][1,2,3]diazaborinine-2(1*H*)-carboxamide (***8***)

Moreover, 3-Formyl-2-thienylboronic acid (0.5 mmol) in acetonitrile (0.10 M) was added to a solution of semicarbazide hydrochloride in acetonitrile (0.1 M), and the resulting mixture was stirred at reflux (90 °C) overnight. Upon completion (LC-MS controlled), the solvent was removed *in vacuo*, and the residue was washed with plenty of water, filtered and dried in a desiccator to furnish the desired compound **8** as pale brown needle-like crystals (42 mg; 43%); mp 314 °C (*dec.*); $\nu_{max}$/$cm^{-1}\;$ (ATR) 3445w (N–H asymmetric), 3190br (N–H symmetric), 1700m (C=O), 1449m (B–O), 1396m (B–N), 1309s (C–B), 672m (B–O); $\delta_{H}$ (600 MHz, acetonitrile-$d_{3}$) 8.15 (1 H, s, γ-C*H*), 7.98 (1 H, d, *J* 5.0, C*H*-Ar), 7.52–7.52 (1 H, d, *J* 5.0, C*H*-Ar); $\delta_{C}$ (151 MHz; acetonitrile-$d_{3}$) 126.7–136.4 (2 × *C*H-Ar and 2 × *C*-Ar), 145.0 (γ-*C*), 163.8 (*C*=O); $\delta_{B}$ (193 MHz, acetonitrile-$d_{3}$) 28.8; LC-MS (retention time: 5.9 min) m/z (ESI) 196 (M^+^ + H, 100%), 373 (2 × M^+^ + H, 6.0); HRMS (ESI) calcd. for C_6_H_6_BN_3_O_2_S [M + H]^+^m/z = 196.0347, found: 196.0347.

### 3.11. 1-Hydroxybenzo[*d*][1,2,3]-diazaborinine-2(1*H*)-carbothioamide (***9***)

Benzodiazaborine **9** was synthesised from 2-FPBA and thiosemicarbazide according to the general procedure [16]. The needle-like crystals were collected, washed with cold water and purified using RP-HPLC (retention time: 10.2–10.5 min) (5.0–50% TFA (0.1% v/v) in MeCN/ TFA (0.1% v/v) in H_2_O over 40 min; 3.0 mL min^−1^, 40 °C) to furnish the desired compound **9** as a white fluffy powder (27 mg; 26%); mp 187 °C (*dec.*); $\nu_{max}$//cm (ATR) 3398m (N–H asymmetric), 3263 (N–H symmetric), 3040w ($C_{{sp}^{3}}$–H), 1586s (C=S), 1297m (C–N), 1255m (C–B); $\delta_{H}$ (600 MHz, acetonitrile-$d_{3}$) 8.15–8.12 (1 H, d, *J* 2.0, γ-C*H*), 7.79–7.72 (1 H, m, C*H*-Ar), 7.60–7.56 (1 H, m, C*H*-Ar), 7.40–7.36 (2 H, m, 2 × C*H*-Ar); $\delta_{C}$ (151 MHz; acetonitrile-$d_{3}$) 128.6–136.6 (4 × *C*H-Ar and 2 × *C*-Ar), 142.4 and 145.9 (γ-*C*H), 178.8 (*C*=S); $\delta_{B}$ (193 MHz, acetonitrile-$d_{3}$) 29.9; LC-MS (retention time: 5.01 min) m/z (ESI) 206 (M^+^ + H, 100%), 224 (M^+^ + H_3_O, 35), 393 (2 × M^+^, 15); HRMS (ESI) calcd. for C_8_H_8_BN_3_OS [M + H]^+^m/z = 206.0554, found: 206.0553.

### 3.12. 1-Hydroxy-4-methylbenzo[*d*][1,2,3]diazaborinine-2(1*H*)-carbothioamide (***10***)

Benzodiazaborine **10** was synthesised from 2-APBA and thiosemicarbazide according to the general procedure [16]. The precipitated powder was washed with cold water and purified using RP-HPLC (retention time: 16.3–16.6 min) (5.0–50% TFA (0.1% v/v) in MeCN/ TFA (0.1% v/v) in H_2_O over 40 min; 3.0 mL min^−1^, 40 °C) to furnish the desired compound **10** as a white powder (61 mg; 56%); mp 249 °C (*dec.*); $\nu_{max}$/$cm^{-1}\;$ (ATR) 3389m (N–H asymmetric), 3251 (N–H symmetric), 1586m (C=S), 1408s ($C_{{sp}^{2}}$=$C_{{sp}^{2}}$), 1303m (C–N); $\delta_{H}$ (600 MHz, acetonitrile-$d_{3}$) 8.17–8.15 (1 H, m, C*H*-Ar), 7.90–7.87 (1 H, m, C*H*-Ar), 7.80–7.76 (1 H, m, C*H*-Ar), 7.70–7.66 (1 H, m, C*H*-Ar), 2.57 (3 H, s, C$H_{3}$); $\delta_{C}$ (151 MHz; acetonitrile-$d_{3}$) 20.2 (*C*H_3_) 126.8–134.9 (4 × *C*H-Ar and 2 × *C*-Ar), 147.6 (γ-*C*), 186.7 (*C*=S); $\delta_{B}$ (193 MHz, acetonitrile-$d_{3}$) 30.0; LC-MS (retention time: 7.37 min) m/z (ESI) 220 (M^+^ + H, 100%), 420 (2 × M^+^ + H, 10); HRMS (ESI) calcd. for C_9_H_10_BN_3_OS [M + H]^+^m/z = 220.0710, found: 220.0710.

### 3.13. (2aS)-4a-Hydroxy-2,2a,4a,8b-tetrahydro-3*H*-4-oxa-1-thia-2a^1^-aza-4a$\lambda^{4}$-borapentaleno[1,6-ab]inden-3-one (***11***)

Heterocycle **11** was synthesised from 2-FPBA and D-cysteine according to the general procedure [16]. The clear reaction mixture was lyophilised, and the residue was purified using RP-HPLC (retention time: 20.1–21.4 min) (5.0–50% TFA (0.1% v/v) in MeCN/ TFA (0.1% v/v) in H_2_O over 40 min; 3.0 mLmin^−1^, 40 °C) to yield the desired compound **11** as a white fluffy powder (83 mg; 71%); mp 310–313 °C (*dec.*); ${\left[\alpha \right]}_{D}^{25}$ -19.6 (c 0.025 in acetonitrile); $\nu_{max}$/$cm^{-1}\;$ (ATR) 3060w ($C_{{sp}^{3}}$–H), 1730s (C=O), 1320m (C–N), 1204m (C–B); $\delta_{H}$ (600 MHz, acetonitrile-$d_{3}$) 7.44–7.42 (1 H, m, C*H*-Ar), 7.31–7.28 (2 H, m, 2 × C*H*-Ar), 7.19–7.17 (1 H, m, C*H*-Ar), 6.16 (1 H, s, δ-C*H*), 4.75–4.72 (1 H, d, ^3^$J_{H\alpha ,H\beta }$ 6.0, α-C*H*), 3.59–3.55 (1 H, ABq, ^1^$J_{H\beta ,H\beta }$ 12.0 and ^3^$J_{H\alpha ,H\beta }$ 6.0, β-C*H*), 3.46–3.42 (1 H, ABq, ^1^$J_{H\beta ,H\beta }$ 12.0 and ^3^$J_{H\alpha ,H\beta }$ 6.0, β-C*H*); $\delta_{C}$ (151 MHz; acetonitrile-$d_{3}$) 37.9 (β-*C*H_2_), 65.6 (δ-*C*H), 73.4 (α-*C*H), 123.1–143.2 (4 × *C*H-Ar and 2 × *C*-Ar), 174.1 (*C*=O); $\delta_{B}$ (193 MHz, acetonitrile-$d_{3}$) 11.4; LC-MS (retention time: 4.58 min) m/z (ESI) 236 (M^+^ + H, 100%), 453 (2 × M^+^ + H, 100); HRMS (ESI) calcd. for C_10_H_10_BNO_3_S [M + H]^+^m/z = 236.0547, found: 236.0545.

### 3.14. (2aR)-4a-Hydroxy-2,2a,4a,8b-tetrahydro-3*H*-4-oxa-1-thia-2a^1^-aza-4a$\lambda^{4}$-borapentaleno[1,6-ab]inden-3-one (***12***)

Heterocycle **12** was synthesised from 2-FPBA and L-cysteine according to the general procedure [16]. The clear reaction mixture was lyophilised, and the residue was purified using RP-HPLC (retention time: 20.1–21.4 min) (5.0–50% TFA (0.1% v/v) in MeCN/ TFA (0.1% v/v) in H_2_O over 40 min; 3.0 mL min^−1^, 40 °C) to yield the desired compound **12** as a white fluffy powder (78 mg; 67%); mp 310–313 °C (*dec.*); ${\left[\alpha \right]}_{D}^{25}$ +5.8 (c 0.025 in acetonitrile); $\nu_{max}$/$cm^{-1}\;$ (ATR) 3060w ($C_{{sp}^{3}}$–H), 1730s (C=O), 1320m (C–N), 1204m (C–B); $\delta_{H}$ (600 MHz, acetonitrile-$d_{3}$) 7.44–7.42 (1 H, m, C*H*-Ar), 7.31–7.28 (2 H, m, 2 × C*H*-Ar), 7.19–7.17 (1 H, m, C*H*-Ar), 6.16 (1 H, s, δ-C*H*), 4.75–4.72 (1 H, d, ^3^$J_{H\alpha ,H\beta }$ 6.0, α-C*H*), 3.59–3.55 (1 H, ABq, ^1^$J_{H\beta ,H\beta }$ 12.0 and ^3^$J_{H\alpha ,H\beta }$ 6.0, β-C*H*), 3.46–3.42 (1 H, ABq, ^1^$J_{H\beta ,H\beta }$ 12.0 and ^3^$J_{H\alpha ,H\beta }$ 6.0, β-C*H*); $\delta_{C}$ (151 MHz; acetonitrile-$d_{3}$) 37.9 (β-*C*H_2_), 65.6 (δ-*C*H), 73.4 (α-*C*H), 123.1–143.2 (4 × *C*H-Ar and 2 × *C*-Ar), 174.1 (*C*=O); $\delta_{B}$ (193 MHz, acetonitrile-$d_{3}$) 11.4; LC-MS (retention time: 4.58 min) m/z (ESI) 236 (M^+^ + H, 100%), 453 (2 × M_+_ + H, 100); HRMS (ESI) calcd. for C_10_H_10_BNO_3_S [M + H]^+^m/z = 236.0547, found: 236.0545.

### 3.15. (2aR)-4a-Hydroxy-2,2-dimethyl-2,2a,4a,8b-tetrahydro-3H-4-oxa-1-thia-2a^1^-aza-4a$\lambda^{4}$-borapentaleno[1,6-ab]inden-3-one (***13***)

Heterocycle **13** was synthesised from 2-FPBA and L-penicillamine according to the general procedure [16]. The clear reaction mixture was lyophilised, and the residue was purified using RP-HPLC (retention time: 17.5–18.3 min) (5.0–50% TFA (0.1% *v*/*v*) in MeCN/ TFA (0.1% *v*/*v*) in H_2_O over 40 min; 3.0 mL min^−1^, 40 °C) to yield the desired compound **13** as a white fluffy powder (59 mg; 45%); mp 250–255 °C (*dec.*); ${\left[\alpha \right]}_{D}^{25}$ +21.3 (c 0.025 in acetonitrile); $\nu_{max}$/$cm^{-1}\;$ (ATR) 2935w ($C_{{sp}^{3}}$–H), 1713s (C=O), 1204s (C–N), 1167vs (C–B); $\delta_{H}$ (600 MHz, acetonitrile-$d_{3}$) 7.42–7.14 (4 H, m, 4 × C*H*-Ar), 6.25 (1 H, s, δ-C*H*), 4.29 (1 H, s, α-C*H*), 1.61 (3 H, s, C$H_{3}$), 1.41 (3 H, s, C$H_{3}$); $\delta_{C}$ (151 MHz; acetonitrile-$d_{3}$) 23.4 and 29.9 (2 × *C*H_3_), 58.7 (β-*C*H_3_), 71.2 (δ-*C*H), 72.1 (α-*C*H), 128.7–143.7 (4 × *C*H-Ar and 2 × *C*-Ar), 171.5 (*C*=O); $\delta_{B}$ (193 MHz, acetonitrile-$d_{3}$) 11.3; LC-MS (retention time: 5.4 min) m/z (ESI) 264 (M^+^ + H, 100%), 509 (2 × M^+^ + H, 100); HRMS (ESI) calcd. for C_12_H_14_BNO_3_S [M + H]^+^m/z = 264.0860, found: 264.0862.

### 3.16. (2aS)-4a-Hydroxy-2,2-dimethyl-2,2a,4a,8b-tetrahydro-3H-4-oxa-1-thia-2a^1^-aza-4a$\lambda^{4}$-borapentaleno[1,6-ab]inden-3-one (***14***)

Heterocycle **14** was synthesised from 2-FPBA and D-penicillamine according to the general procedure [16]. The clear reaction mixture was lyophilised, and the residue was purified using RP-HPLC (retention time: 17.1–17.9 min) (5.0–50% TFA (0.1% *v*/*v*) in MeCN/ TFA (0.1% *v*/*v*) in H_2_O over 40 min; 3.0 mL min^−1^, 40 °C) to yield the desired compound **14** as a white fluffy powder (67 mg; 51%); mp 250–255 °C (*dec.*); ${\left[\alpha \right]}_{D}^{25}$ –16.4 (c 0.025 in acetonitrile); $\nu_{max}$/$cm^{-1}\;$ (ATR) 2935w ($C_{{sp}^{3}}$–H), 1713s (C=O), 1204s (C–N), 1167vs (C–B); $\delta_{H}$ (600 MHz, acetonitrile-$d_{3}$) 7.42–7.14 (4 H, m, 4 × C*H*-Ar), 6.25 (1 H, s, δ-C*H*), 4.29 (1 H, s, α-C*H*), 1.61 (3 H, s, C$H_{3}$), 1.41 (3 H, s, C$H_{3}$); $\delta_{C}$ (151 MHz; acetonitrile-$d_{3}$) 23.4 and 29.9 (2 × *C*H_3_), 58.7 (β-*C*H_3_), 71.2 (δ-*C*H), 72.1 (α-*C*H), 128.7–143.7 (4 × *C*H-Ar and 2 × *C*-Ar), 171.5 (*C*=O); $\delta_{B}$ (193 MHz, acetonitrile-$d_{3}$) 11.3; LC-MS (retention time: 5.4 min) m/z (ESI) 264 (M^+^ + H, 100%), 509 (2 × M^+^ + H, 100); HRMS (ESI) calcd. for C_12_H_14_BNO_3_S [M + H]^+^m/z = 264.0860, found: 264.0862.

### 3.17. (2aS)-4a-Hydroxy-8b-methyl-2,2a,4a,8b-tetrahydro-3H-4-oxa-1-thia-2a^1^-aza-4a$\lambda^{4}$-borapentaleno[1,6-ab]inden-3-one (***15***)

Heterocycle **15** was synthesised from 2-APBA and D-cysteine according to the general procedure [16]. The clear reaction mixture was lyophilised, and the residue was purified using RP-HPLC (retention time: 19.3–20.5 min) (5.0–50% TFA (0.1% *v*/*v*) in MeCN/ TFA (0.1% *v*/*v*) in H_2_O over 40 min; 3.0 mL min^−1^, 40 °C) to yield the desired compound **15** as a white fluffy powder (77 mg; 62%); mp 200 °C (*dec.*); ${\left[\alpha \right]}_{D}^{25}$ –14.4 (c 0.025 in acetonitrile); $\nu_{max}$/$cm^{-1}\;$ (ATR) 3416w (O–H H-bonding), 1715s (C=O), 1260m (C–N), 1227m (C–B); $\delta_{H}$ (600 MHz, acetonitrile-$d_{3}$) 7.42–7.40 (1 H, d, *J* 6.0, C*H*-Ar), 7.34–7.28 (2 H, m, 2 × C*H*-Ar), 7.24–7.22 (1 H, d, *J* 6.0, C*H*-Ar), 4.79–4.77 (1 H, ABX, ^3^$J_{H\alpha ,H\beta_{1}}$ 8.0 and ^3^$J_{H\alpha ,H\beta_{2}}$ 4.0, α-C*H*), 3.65–3.62 (1 H, ABq, ^1^$J_{H\beta_{1},H\beta_{2}}$ 12.0 and _3_$J_{H\alpha ,H\beta_{1}}$ 8.0, $\beta_{1}$-C*H*), 3.42–3.39 (1 H, ABq, ^1^$J_{H\beta_{1},H\beta_{2}}$ 12.0 and ^3^$J_{H\alpha ,H\beta_{2}}$ 4.0, $\beta_{2}$-C*H*), 2.01 (3 H, s, C$H_{3}$); $\delta_{C}$ (151 MHz; acetonitrile-$d_{3}$) 29.1 (*C*H_3_), 35.7 (β-*C*H_2_) 66.8 (δ-*C*), 85.6 (α-*C*H), 122.0–147.9 (4 × *C*H-Ar and 2 × *C*-Ar), 174.0 (*C*=O); $\delta_{B}$ (193 MHz, acetonitrile-$d_{3}$) 11.1; LC-MS (retention time: 3.59 min) m/z (ESI) 250 (M^+^ + H, 100%), 521 (2 × M^+^ + Na, 20); HRMS (ESI) calcd. for C_11_H_12_BNO_3_S [M + H]^+^m/z = 250.0704, found: 250.0701.

### 3.18. (2aR)-4a-Hydroxy-8b-methyl-2,2a,4a,8b-tetrahydro-3H-4-oxa-1-thia-2a^1^-aza-4a$\lambda^{4}$-borapentaleno[1,6-ab]inden-3-one (***16***)

Heterocycle **16** was synthesised from 2-APBA and L-cysteine according to the general procedure [16]. The clear reaction mixture was lyophilised, and the residue was purified using RP-HPLC (retention time: 19.8–20.9 min) (5.0–50% TFA (0.1% *v*/*v*) in MeCN/ TFA (0.1% *v*/*v*) in H_2_O over 40 min; 3.0 mL min^−1^, 40 °C) to yield the desired compound **16** as a white fluffy powder (77 mg; 62%); mp 200 °C (*dec.*); ${\left[\alpha \right]}_{D}^{25}$ +2.3 (c 0.025 in acetonitrile); $\nu_{max}$/$cm^{-1}\;$ (ATR) 3416w (O–H H-bonding), 1715s (C=O), 1260m (C–N), 1227m (C–B); $\delta_{H}$ (600 MHz, acetonitrile-$d_{3}$) 7.42–7.40 (1 H, d, *J* 6.0, C*H*-Ar), 7.34–7.28 (2 H, m, 2 × C*H*-Ar), 7.24–7.22 (1 H, d, *J* 6.0, C*H*-Ar), 4.79–4.77 (1 H, ABX, ^3^$J_{H\alpha ,H\beta_{1}}$ 8.0 and ^3^$J_{H\alpha ,H\beta_{2}}$ 4.0, α-C*H*), 3.65–3.62 (1 H, ABq, ^1^$J_{H\beta_{1},H\beta_{2}}$ 12.0 and ^3^$J_{H\alpha ,H\beta_{1}}$ 8.0, $\beta_{1}$-C*H*), 3.42–3.39 (1 H, ABq, ^1^$J_{H\beta_{1},H\beta_{2}}$ 12.0 and ^3^$J_{H\alpha ,H\beta_{2}}$ 4.0, $\beta_{2}$-C*H*), 2.01 (3 H, s, C$H_{3}$); $\delta_{C}$ (151 MHz; acetonitrile-$d_{3}$) 29.1 (*C*H_3_), 35.7 (β-*C*H_2_) 66.8 (δ-*C*), 85.6 (α-*C*H), 122.0–147.9 (4 × *C*H-Ar and 2 × *C*-Ar), 174.0 (*C*=O); $\delta_{B}$ (193 MHz, acetonitrile-$d_{3}$) 11.1; LC-MS (retention time: 3.59 min) m/z (ESI) 250 (M^+^ + H, 100%), 521 (2 × M^+^ + Na, 20); HRMS (ESI) calcd. for C_11_H_12_BNO_3_S [M + H]^+^m/z = 250.0704, found: 250.0701.

### 3.19. (2aS)-4a-Hydroxy-2,2,8b-trimethyl-2,2a,4a,8b-tetrahydro-3H-4-oxa-1-thia-2a^1^-aza-4a$\lambda^{4}$-borapentaleno[1,6-ab]inden-3-one (***17***)

Heterocycle **17** was synthesised from 2-APBA and D-penicillamine according to the general procedure [16]. The clear reaction mixture was lyophilised, and the residue was purified using RP-HPLC (retention time: 22.7–24.1 min) (5.0–50% TFA (0.1% *v*/*v*) in MeCN/ TFA (0.1% *v*/*v*) in H_2_O over 40 min; 3.0 mL min^−1^, 40 °C) to yield the desired compound **17** as a white powder (54 mg; 39%); mp 201 °C (*dec.*); ${\left[\alpha \right]}_{D}^{25}$ –4.5 (c 0.015 in acetonitrile); $\nu_{max}$/$cm^{-1}\;$ (ATR) 3040w ($C_{{sp}^{3}}$–H), 1723s (C=O), 1281m (C–N), 1242m (C–B); $\delta_{H}$ (600 MHz, acetonitrile-$d_{3}$) 7.38–7.28 (4 H, m, 4 × C*H*-Ar), 4.47 (1 H, s, α-C*H*), 1.94 (3 H, s, C$H_{3}$), 1.55 and 0.83 (6 H, s, 2 × C$H_{3}$); $\delta_{C}$ (151 MHz; acetonitrile-$d_{3}$) 26.5 (2 × *C*H_3_), 29.9 (*C*H_3_), 56.4 (β-*C*), 76.2 (δ-*C*), 83.5 (α-*C*H), 123.0–151.6 (4 × *C*H-Ar and 2 × *C*-Ar), 171.4 (*C*=O); $\delta_{B}$ (193 MHz, acetonitrile-$d_{3}$) 11.5; LC-MS (retention time: 4.47 min) m/z (ESI) 278 (M^+^ + H, 100%), 537 (2 × M^+^, 40), 577 (2 × M^+^ + MeCN, 20); HRMS (ESI) calcd. for C_13_H_16_BNO_3_S [M + H]^+^m/z = 278.1017, found: 278.1014.

### 3.20. (2aR)-4a-Hydroxy-2,2,8b-trimethyl-2,2a,4a,8b-tetrahydro-3H-4-oxa-1-thia-2a^1^-aza-4a$\lambda^{4}$-borapentaleno[1,6-ab]inden-3-one (***18***)

Heterocycle **18** was synthesised from 2-APBA and L-penicillamine according to the general procedure [16]. The clear reaction mixture was lyophilised, and the residue was purified using RP-HPLC (retention time: 22.4–23.9 min) (5.0–50% TFA (0.1% *v*/*v*) in MeCN/ TFA (0.1% *v*/*v*) in H2_O_ over 40 min; 3.0 mL min^−1^, 40 °C) to yield the desired compound **18** as a white powder (37 mg; 27%); mp 201 °C (*dec.*); ${\left[\alpha \right]}_{D}^{25}$ +8.3 (c 0.015 in acetonitrile); $\nu_{max}$/$cm^{-1}\;$ (ATR) 3040w ($C_{{sp}^{3}}$–H), 1723s (C=O), 1281m (C–N), 1242m (C–B); $\delta_{H}$ (600 MHz, acetonitrile-$d_{3}$) 7.38–7.28 (4 H, m, 4 × C*H*-Ar), 4.47 (1 H, s, α-C*H*), 1.94 (3 H, s, C$H_{3}$), 1.55 and 0.83 (6 H, s, 2 × C$H_{3}$); $\delta_{C}$ (151 MHz; acetonitrile-$d_{3}$) 26.5 (2 × *C*H_3_), 29.9 (*C*H_3_), 56.4 (β-*C*), 76.2 (δ-*C*), 83.5 (α-*C*H), 123.0–151.6 (4 × *C*H-Ar and 2 × *C*-Ar), 171.4 (*C*=O); $\delta_{B}$ (193 MHz, acetonitrile-$d_{3}$) 11.5; LC-MS (retention time: 4.47 min) m/z (ESI) 278 (M^+^ + H, 100%), 537 (2 × M^+^, 40), 577 (2 × M^+^ + MeCN, 20); HRMS (ESI) calcd. for C_13_H_16_BNO_3_S [M + H]^+^m/z = 278.1017, found: 278.1014.

### 3.21. (E)-2-(Thiophen-3-ylmethylene)hydrazine-1-carboxamide (***19***)

Carboxamide **19** was synthesised from 3-FTBA and semicarbazide according to the general procedure [16]. The precipitated powder was washed with cold water and lyophilised to furnish the desired compound **19** as an off-white powder (53 mg; 54%); mp 220 °C (*dec.*) (lit. [18] 233–234 °C); $\nu_{max}$/$cm^{-1}\;$ (ATR) 3453w (N–H asymmetric), 3106br (N–H symmetric), 1684s (C=O), 1598s ($C_{{sp}^{2}}$–$C_{{sp}^{2}}$), 1170m (C–N); $\delta_{H}$ (600 MHz, acetonitrile-$d_{3}$) 7.90 (1 H, s, γ-C*H*), 7.62–7.61 (1 H, m, C*H*-Ar), 7.50–7.49 (1 H, m, C*H*-Ar), 7.42–7.41 (1 H, m, C*H*-Ar); $\delta_{C}$ (151 MHz; acetonitrile-$d_{3}$) 125.3–127.6 (3 × *C*H-Ar and 1 × *C*-Ar), 138.0 (γ-*C*H), 138.7 (*C*=O); LC-MS (retention time: 5.1 min) m/z (ESI) 170 (M^+^ + H, 100%); HRMS (ESI) calcd. for C_6_H_7_N_3_OS [M + H]^+^m/z = 170.0383, found: 170.0382.

### 3.22. Propane-1-sulfonohydrazide (***20***)

To a chilled solution of hydrazine monohydrate (4.9 mL, 0.10 mol) in anhydrous THF (20 mL) was added 1-propanesulfonyl chloride (1.1 mL, 10 mmol) in anhydrous THF (10 mL) at 5.0 °C dropwise. The thus obtained clear reaction mixture was stirred at 10–20 °C for 18 h. Upon completion (LC-MS controlled), the clear reaction mixture was extracted with EtOAc (5.0 × 50 mL), the combined organic extracts were washed with aqueous sodium hypochlorite 5.0% *w/w* (100 mL), dried over Na_2_SO_4_, filtered and the solvent was removed *in vacuo*. The crude product was purified by flash column chromatography to yield the desired compound **20** as a white solid (1.3 g; 94%); R_f_ 0.40 (10% MeOH in CH_2_Cl_2_); mp 45 °C (lit. [19] 40–41 °C); $\nu_{max}$/$cm^{-1}\;$ (ATR) 3303m (N–H asymmetric), 3243m (N–H symmetric), 1619s (C=O); $\delta_{H}$ (400 MHz, CDCl_3_) 3.12–3.04 (2 H, m, α-C$H_{2}$), 1.91–1.80 (2 H, m, β-C$H_{2}$), 1.07 (3 H, t, *J* 7.5, γ-C$H_{3}$); $\delta_{C}$ (101 MHz, CDCl_3_) 13.1 (γ-*C*H_3_), 17.1, (β-*C*H_2_), 51.2 (α-*C*H_2_); LC-MS (retention time: 1.94 min) m/z (ESI) 139 (M^+^ + H, 100%).

### 3.23. 6-Methylthieno[3,2-d][1,2,3]diazaborinin-1(2H)-ol (***21***)

A solution of bread thiophene (40 mg, 0.30 mmol) in dichloroethane (5.0 mL) was added to hydrazide 20 dropwise, to which was added BBr_3_ (0.10 mL, 1.0 mmol) in dichloroethane (0.30 mL) followed by AlCl_3_ (4.0 mg, 30 μmol) dissolved in dichloroethane (2.0 mL) under argon. The thus obtained yellow reaction mixture was stirred at reflux under argon for 20 min. The reaction mixture was cooled in an ice bath and poured into ice water (3.0 mL); the organic phase was separated, washed with deionised water (2.0 × 0.50 mL) and extracted with 1.0 M aqueous sodium hydroxide (3.0 × 2.0 mL). The aqueous extracts were collected and conc. HCl added until acidic to Congo red. The acidified aqueous extracts were extracted with CH_2_Cl_2_ (2.0 × 5.0 mL), dried over Na_2_SO_4_, and the solvent was removed *in vacuo* to yield a yellow wax. The residue was purified by flash chromatography to furnish the desired compound **21** as a green wax (30 mg; 60%); R_f_ 0.40 (10% MeOH in CH_2_Cl_2_); $\delta_{H}$ (600 MHz, acetonitrile-$d_{3}$) 9.95 (1 H, s, C*H*=N), 8.01 (1 H, s, C=C*H*=C), 2.57 (3 H, d,*J* 1.0, C$H_{3}$); $\delta_{C}$ (151 MHz; acetonitrile-$d_{3}$) 29.6 (*C*H_3_), 127.2–135.2 (1 × *C*H-Ar and 3 × *C*-Ar), 186.5 (*C*H=N); $\delta_{B}$ (193 MHz, acetonitrile-$d_{3}$) 26.3; LC-MS (retention time: 4.7 min) *m*/*z* (ESI) 167 (M^+^ + H, 100%).

### 3.24. (E)-N^′^-((5-Methylthiophen-2-yl)methylene)propane-1-sulfonohydrazide (***22***)

To a solution of bread thiophene (0.78 mL, 7.2 mmol) and hydrazide **20** (1.0 g, 7.2 mmol) in EtOH (10 mL) was added glacial AcOH (6 drops), and the resulting brown reaction mixture was stirred at ambient temperature for 1.0 h. Upon completion (LC-MS controlled), the solvent was removed in vacuo, and the residue was purified by flash column chromatography to furnish the desired compound **22** as a yellow solid (1.6 g; 94%); R*_f_ *0.35 (7% MeOH in CH_2_Cl_2_); mp 108 °C (lit. [20] 109–110 °C); $\nu_{max}$/$cm^{-1}\;$ (ATR) 3186m (N–H), 1148vs (S=O), 945s (S–O); $\delta_{H}$ (400 MHz, CDCl_3_) 7.95 (1 H, s, γ-CH), 7.75 (1 H, s, α-NH), 7.06 (1 H, d, J 3.5, CH-Ar), 6.75–6.66 (1 H, m, CH-Ar), 3.33–3.15 (2 H, m, α-C$H_{2}$), 2.49 (3 H, d, J 1.0, C$H_{3}$), 1.97–1.79 (2 H, m, β-C$H_{2}$), 1.07 (3 H, t, J 7.5, γ-C$H_{3}$); $\delta_{C}$ (101 MHz, CDCl_3_) 13.0 (γ-CH_3_), 15.7 (β-CH_2_), 17.0 (CH_3_), 52.7 (α-CH_2_), 125.8 and 131.1 (CH-Ar), 135.4 (C-Ar), 143.7 (γ-CH), 144.5 (C-Ar); LC-MS (retention time: 7.13 min) m/z (ESI) 247 (M^+^ + H, 100%); HRMS (ESI) calcd. for C_9_H_15_N_2_O_2_S_2_ [M + H]^+^m/z = 247.0570, found: 247.0571.

### 3.25. 6-Methyl-2-(propylsulfonyl)thieno[3,2-d][1,2,3]diazaborinin-1(2H)-ol (***23***)

To a solution of FeCl_3_ (60 mg, 0.37 mmol) in dry dichloroethane was added hydrazone **22** (0.90 g, 3.7 mmol) in dry dichloroethane (50 mL) and BBr_3_ (1.1 mL, 11 mmol) in dry dichloroethane (3.0 mL) simultaneously under argon with vigorous stirring at 70 °C for 15 min. The orange reaction mixture was cooled in an ice bath and poured into ice water (30 mL). The subsequent reaction mixture was washed with deionised water (30 mL), and the organic phase was extracted with 1.0 M aqueous sodium hydroxide (3.0 × 30 mL). The aqueous extracts were combined, and conc. HCl added until a cloudy mixture was obtained (pH ≈ 2–3). The thus obtained colloidal was extracted with CH_2_Cl_2_ (4.0 × 100 mL), dried over Na_2_SO_4_, and the solvent was removed *in vacuo*. The green crude wax was purified by flash column chromatography to furnish the desired compound **23** as a white powder (0.72 g; 72%); R*_f_* 0.30 (5% MeOH in CH_2_Cl_2_); mp 92–94 °C (*dec.*) (lit. [21] 85–86 °C); $\nu_{max}$/$cm^{-1}\;$ (ATR) 3200–2800br (O–H H-bonding), 1361m and 1136s (S=O), 900vs (S–O); $\delta_{H}$ (400 MHz, acetonitrile-$d_{3}$) 8.19 (1 H, s, γ-C*H*), 7.26 (1 H, t, *J* 1.0, C*H*-Ar), 3.48–3.42 (2 H, m, α-C$H_{2}$), 2.59 (3 H, d, *J* 1.0, C$H_{3}$), 1.71 (2 H, dq, *J* 15.0 and 7.5, β-C$H_{2}$), 0.95 (3 H, t, *J* 7.5, γ-C$H_{3}$); $\delta_{C}$R (101 MHz, acetonitrile-$d_{3}$) 12.4 (γ-*C*H_3_), 15.1 (β-*C*H_2_), 17.3 (*C*H_3_), 53.8 (α-*C*H_2_), 128.2 (*C*H-Ar), 136.8 (γ-*C*H), 148.2 (*C*-Ar); $\delta_{B}$ (193 MHz, acetonitrile-$d_{3}$) 27.2; LC-MS (retention time: 7.25 min) m/z (ESI) 273 (M^+^ + H, 100%); HRMS (ESI) calcd. for C_9_H_14_BN_2_O_3_S_2_ [M + H]^+^m/z = 273.0533, found: 273.0534.

## 4. Conclusions

Gao and coworkers [10] have reported that on reacting 2-FPBA and 2-APBA with L-cysteine in 0.10 M phosphate-buffered saline (PBS) buffer at physiological pH, bicyclic or tricyclic heterocycles form, which led to the survey of an array of borazaros and oxazaborons described herein (Figure 3); however, in this work, such reactions exclusively furnished tetracyclic and heteroaromatic structures. Whilst almost all the diazaborines as well as boronate ester **12** described in this work have, one way or another, been reported in the literature, by all means, this work is the first of its kind to delineate a ‘*tetracyclic caged*’ structure for novel boronate esters **11** and **13**–**18**, and report full chemical characterisation, *i.e.*, mp, optical rotation, IR, NMR (^1^H, ^13^C and ^11^B *cf.* Supplementary Materials), LC-MS and HRMS of the aforementioned benzodiazaborines, diazaborines and novel boronate esters for which, so far as this work is concerned, there are no known precedents, and the specific applications and the broad implications of these intriguing heterocycles may, hopefully, be of interest to the synthetic and medicinal chemist alike.

## Figures and Tables

**Table 1:** ^1^H, ^13^C, ^11^B and IR Data for 2-FPBA, Diazaborines & Boronate Esters.

Entry	**^11^**B ^∞^	(C*H*O or γ-C*H* or δ-C*H*) ^¶^	(*C*HO or γ-*C* or δ-*C*) ^♣^	IR: C=O ^♠^
2-FPBA	29.9	10.05	197.0	
1	29.8	8.08	142.7	1673s
2	29.8		146.9	1687s
3	27.7	7.96	140.4	
4	16.2 ^•^	8.35 ^•^	175.5 ^•^	1682s
5	28.3			1683s
6	28.6	8.20	140.0	1698s
7	28.4		142.4	1699s
8	28.8	8.15	145.0	1700s
9	29.9	8.13	144.0	
10	30.0		147.6	
11 ^★^	11.4	6.16	65.6	1730s
13 ^★^	11.3	6.25	71.2	1713s
15 ^★^	11.1		66.8	1715s
17 ^★^	11.5		76.2	1723s

^★^; Boronate esters **11**, **13**, **15** and **17** are enantiomers of **12**, **14**, **16** and **18**, respectively, with identical spectroscopic data. ^∞^; 193 MHz, acetonitrile-$d_{3}$. ^¶^; 600 MHz, acetonitrile-$d_{3}$. ^♣^; 151 MHz, acetonitrile-$d_{3}$. ^•^; benzodiazaborine **4** is much less soluble in acetonitrile-$d_{3}$; its NMR spectra were recorded in methanol-$d_{4}$ instead. ^♠^; $\nu_{max}$/$cm^{-1}\;$ (ATR), s stands for sharp.

## Data Availability

The original contributions presented in this study are included in the article alongside Supplementary Materials, which can be downloaded at: https://www.mdpi.com/article/10.3390/molecules29214998/s1; further enquiries can be directed to the corresponding author.

## Supplementary Materials

The following supporting information can be downloaded at: https://www.mdpi.com/article/10.3390/molecules29214998/s1, Figures S1–S19: ^1^H, ^13^C and ^11^B NMR spectra (where applicable) of the diazaborines & boronate esters.
