# Supplementary material for: 11B NMR Together with Infrared Spectroscopy Provides Insight into Structural Elucidation of Quadrivalent Diazaborines & Cyclic Boronate Esters: Intriguing & Little-Explored
Source: Molecules. 2024 Oct 22;29(21):4998. doi: 10.3390/molecules29214998 (PMC11547293; doi:10.3390/molecules29214998)

Supplementary Information

^{11}B NMR Together with IR Spectroscopy Provides Insight into Structural Elucidation of Quadrivalent Diazaborines & Cyclic Boronate Esters: Intriguing & Little-explored

Ashley L. Dey,❖,✉

❖ Chemistry Research Laboratory, Department of Chemistry, University of Oxford, 12 Mansfield Road, Oxford, OX1 3TA, United Kingdom

✉ uccaald@ucl.ac.uk

Table of Contents

^{1}H, ^{13}C & ^{11}B NMR Spectra S1

1-Hydroxybenzo[*d*][1,2,3]diazaborinine-2(1*H*)-carboxamide (1) S1

1-Hydroxy-4-methylbenzo[*d*][1,2,3]diazaborinine-2(1*H*)-carboxamide (2) S2

Benzo[*d*][1,2,3]diazaborinin-1(2*H*)-ol (3) S3

8*H*-benzo[4,5][1,2,3]diazaborinino[3,2-*b*]benzo[4,5][1,2,3]diazaborinino[2,3-e][1,3,5,2,6]oxadiazadiborinin-8-one (4) S4

5,11-Dimethyl-8*H*-benzo[4,5][1,2,3]diazaborinino[3,2-*b*]benzo[4,5][1,2,3]diazaborinino[2,3-e][1,3,5,2,6]oxadiazadiborinin-8-one (5) S5

1-Hydroxythieno[3,2-*d*][1,2,3]diazaborinine-2(1*H*)-carboxamide (6) S6

1-Hydroxy-4-methylthieno[3,2-*d*][1,2,3]diazaborinine-2(1*H*)-carboxamide (7) S7

1-Hydroxythieno[2,3-*d*][1,2,3]diazaborinine-2(1*H*)-carboxamide (8) S8

1-Hydroxybenzo[*d*][1,2,3]diazaborinine-2(1*H*)-carbothioamide (9) S9

1-Hydroxy-4-methylbenzo[*d*][1,2,3]diazaborinine-2(1*H*)-carbothioamide (10) S10

(2a*S*)-4a-Hydroxy-2,2a,4a,8b-tetrahydro-3*H*-4-oxa-1-thia-2a^{1}-aza-4aλ^{4}-borapentaleno[1,6-*ab*]inden-3-one (11) S11

(±)-4a-Hydroxy-2,2-dimethyl-2,2a,4a,8b-tetrahydro-3*H*-4-oxa-1-thia-2a^{1}-aza-4aλ^{4}-borapentaleno[1,6-*ab*]inden-3-one (13) & (14) S12

(2a*S*)-4a-Hydroxy-8b-methyl-2,2a,4a,8b-tetrahydro-3*H*-4-oxa-1-thia-2a^{1}-aza-4aλ^{4}-borapentaleno[1,6-*ab*]inden-3-one (15) S13

(2a*S*)-4a-Hydroxy-2,2,8b-trimethyl-2,2a,4a,8b-tetrahydro-3*H*-4-oxa-1-thia-2a^{1}-aza-4aλ^{4}-borapentaleno[1,6-*ab*]inden-3-one (17) S14

(*E*)-2-(Thiophen-3-ylmethylene)hydrazine-1-carboxamide (19) S15

Propane-1-sulfonohydrazide (20) S16

6-Methylthieno[3,2-*d*][1,2,3]diazaborinin-1(2*H*)-ol (21) S17

(*E*)-*N'*-((5-Methylthiophen-2-yl)methylene)propane-1-sulfonohydrazide (22) S18

6-Methyl-2-(propylsulfonyl)thieno[3,2-*d*][1,2,3]diazaborinin-1(2*H*)-ol (23) S19

^{1}H, ^{13}C & ^{11}B NMR Spectra

1-Hydroxybenzo[*d*][1,2,3]diazaborinine-2(1*H*)-carboxamide (1)

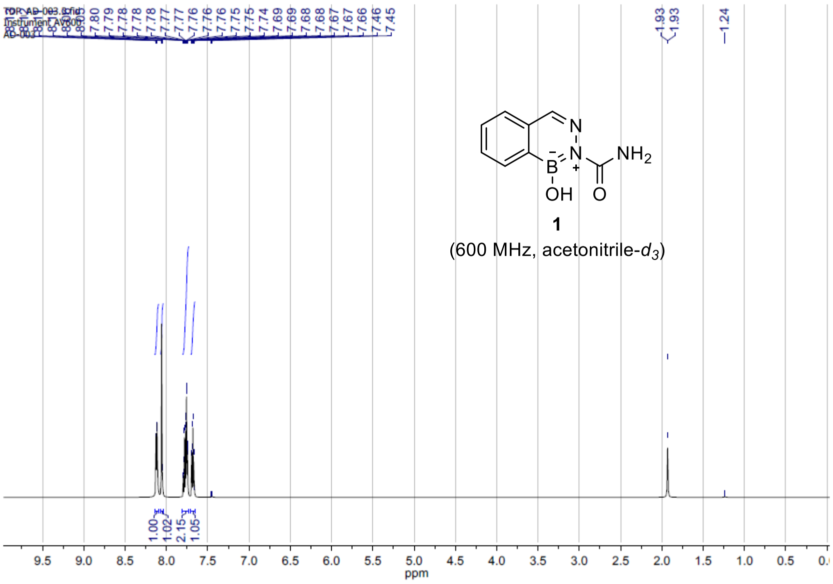

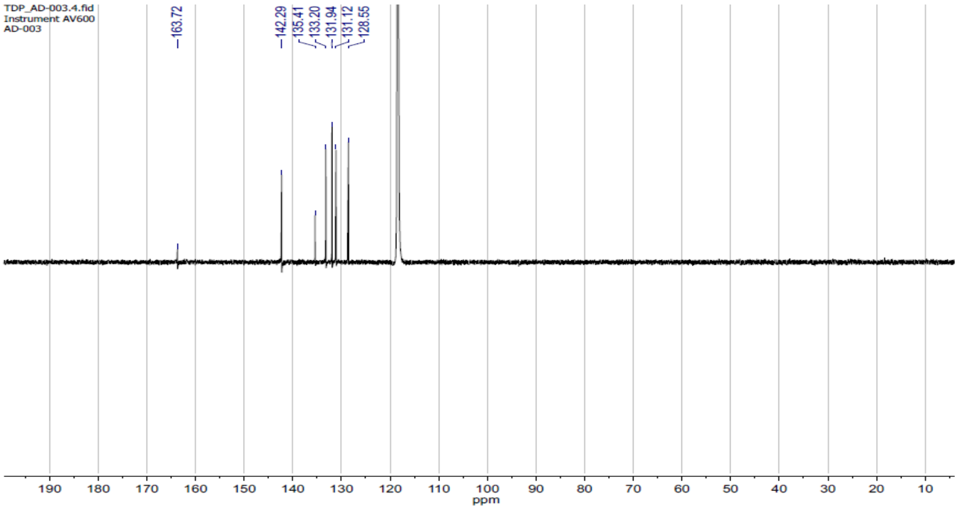

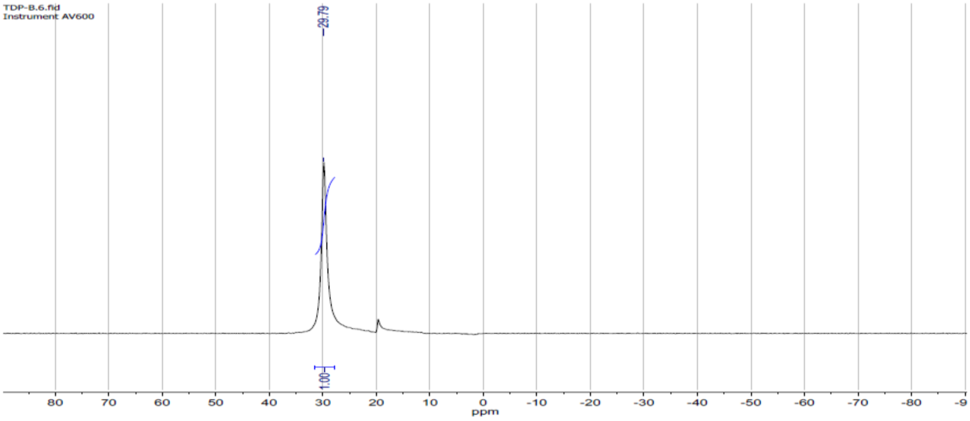

1-Hydroxy-4-methylbenzo[*d*][1,2,3]diazaborinine-2(1*H*)-carboxamide (2)

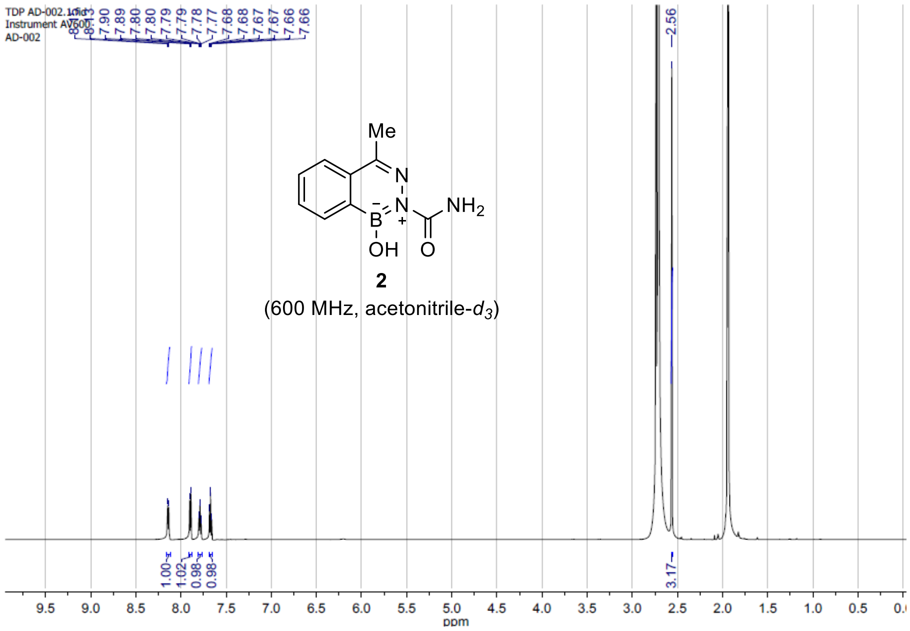

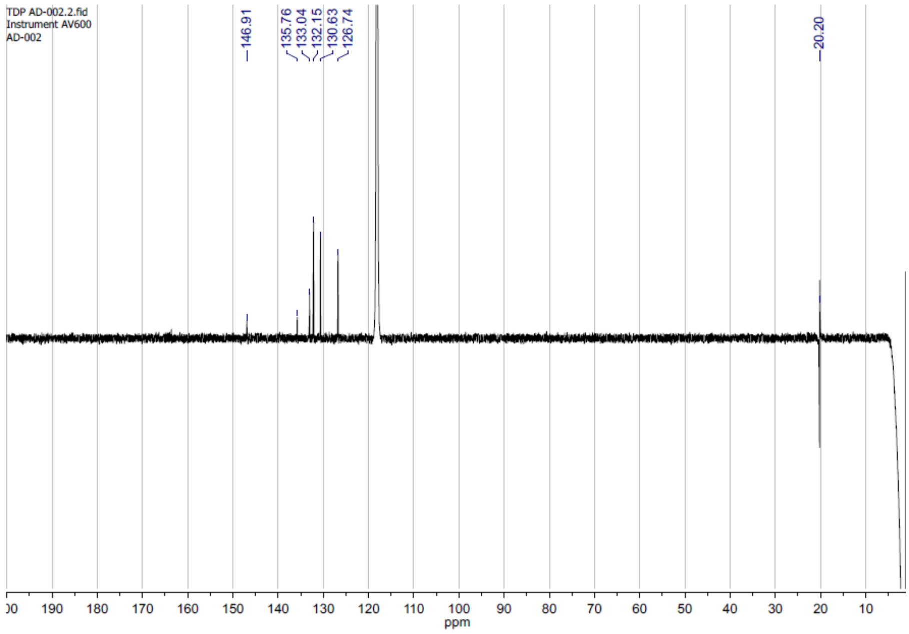

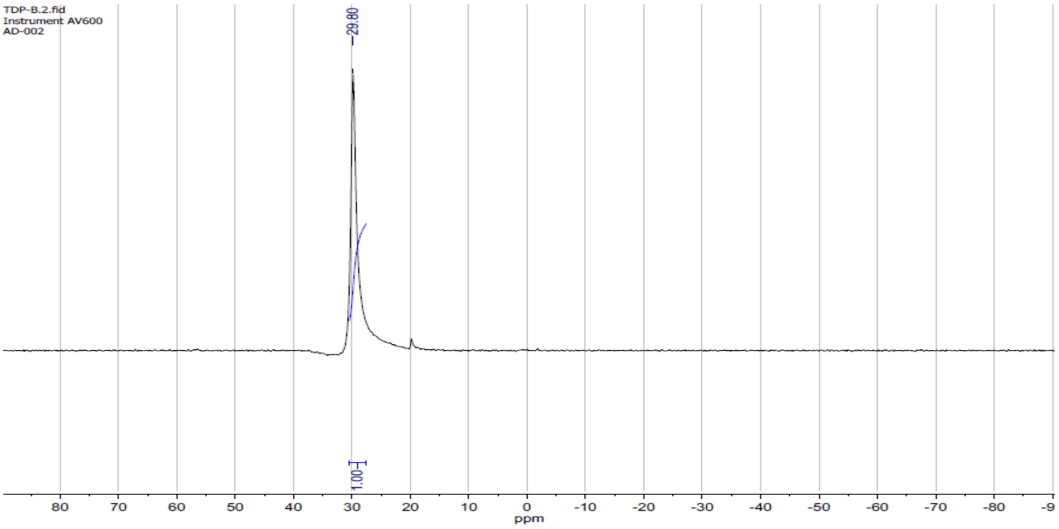

Benzo[*d*][1,2,3]diazaborinin-1(2*H*)-ol (3)

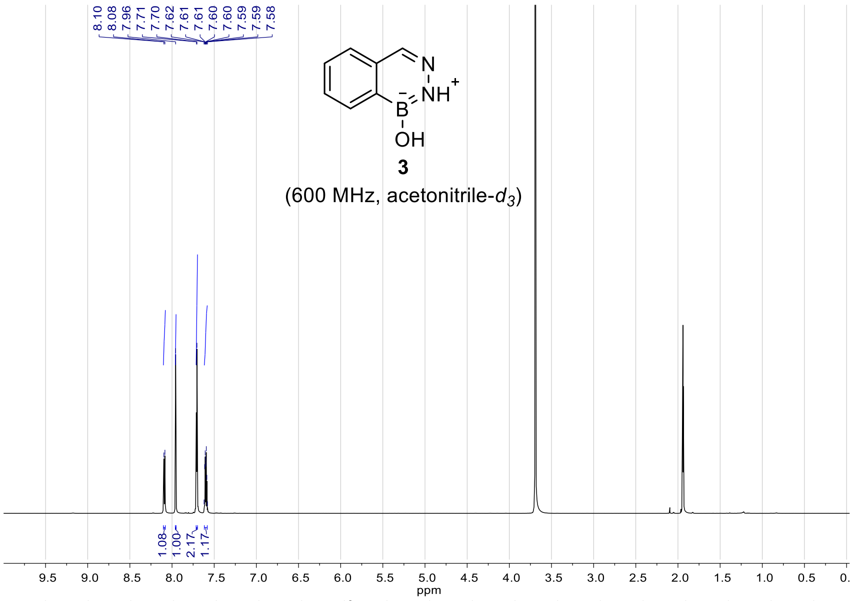

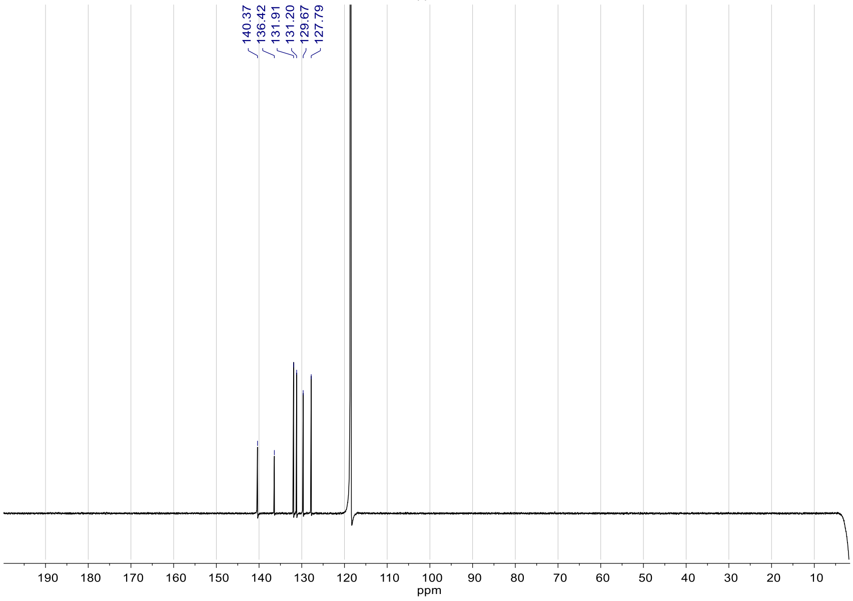

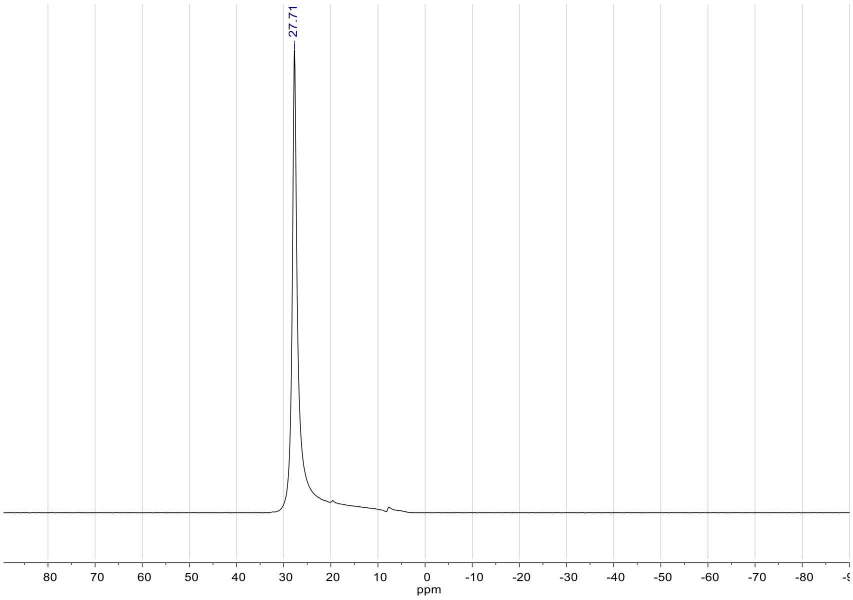

8*H*-benzo[4,5][1,2,3]diazaborinino[3,2-*b*]benzo[4,5][1,2,3]diazaborinino[2,3-e][1,3,5,2,6]oxadiazadiborinin-8-one (4)

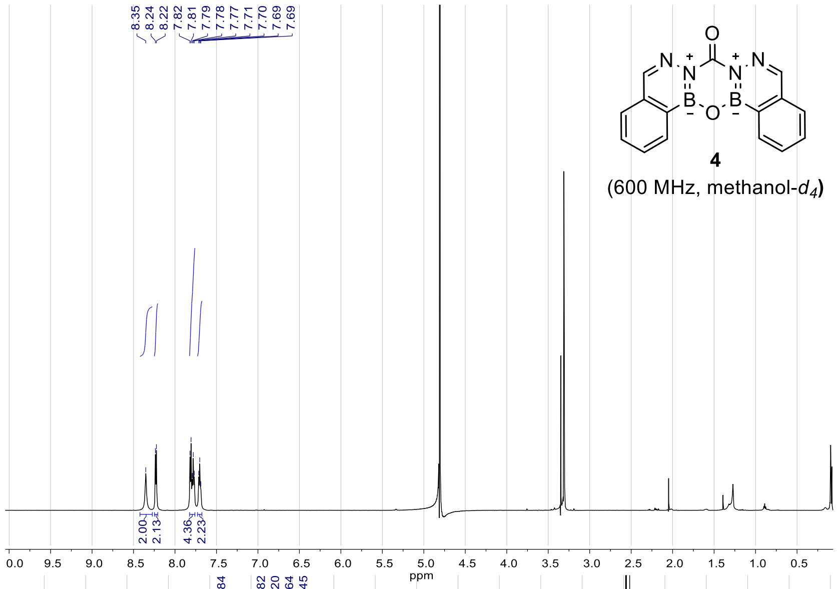

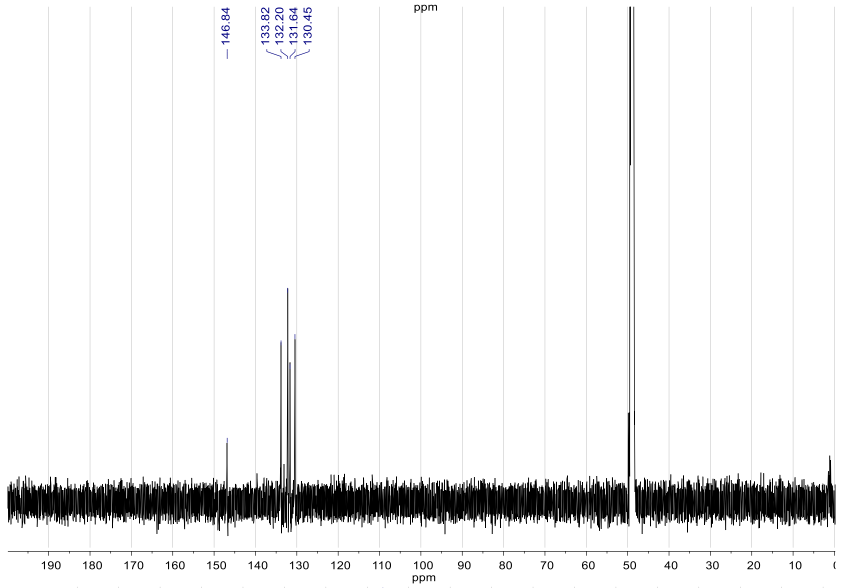

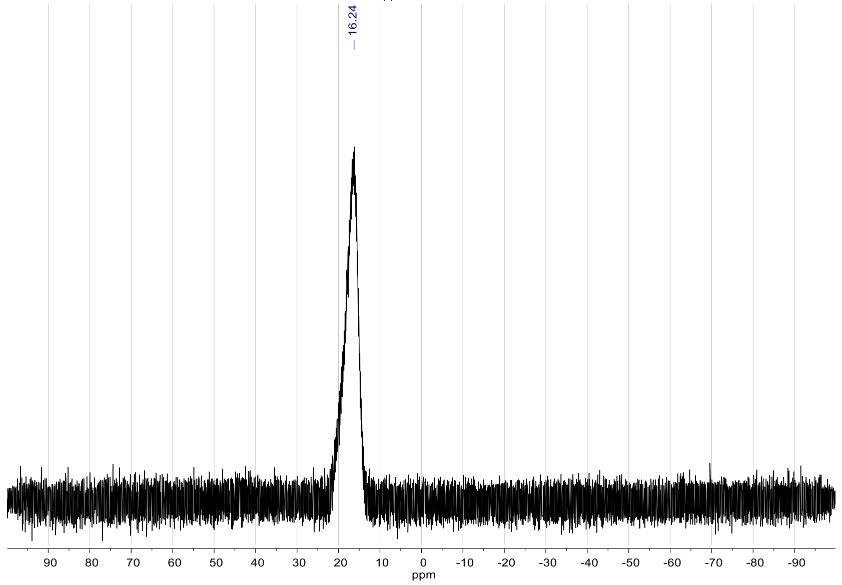

5,11-Dimethyl-8*H*-benzo[4,5][1,2,3]diazaborinino[3,2-*b*]benzo[4,5][1,2,3]diazaborinino[2,3-e][1,3,5,2,6]oxadiazadiborinin-8-one (5)

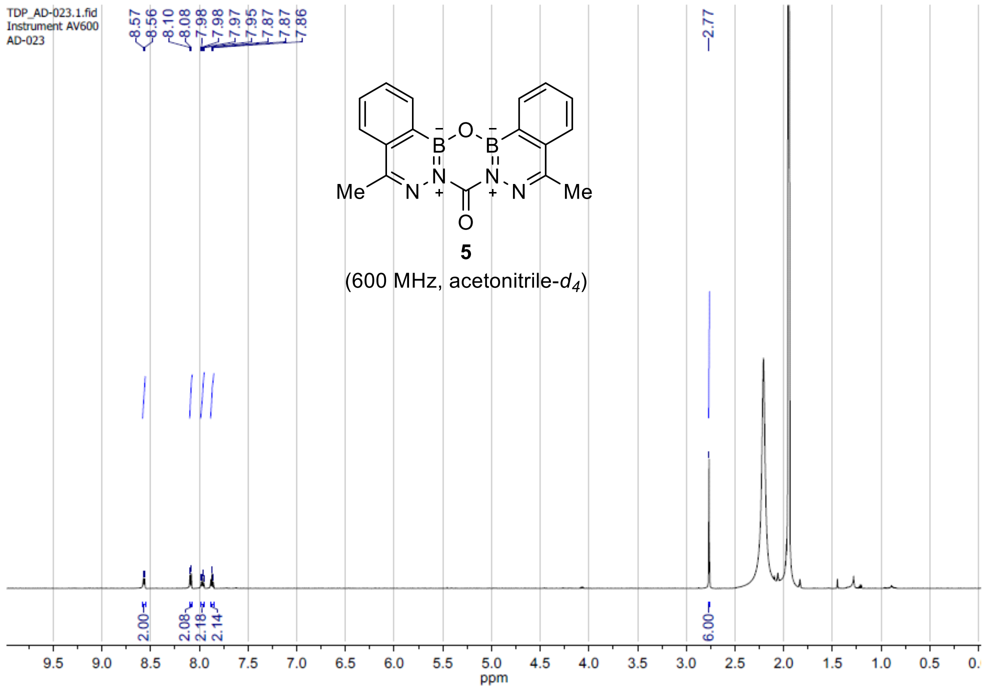

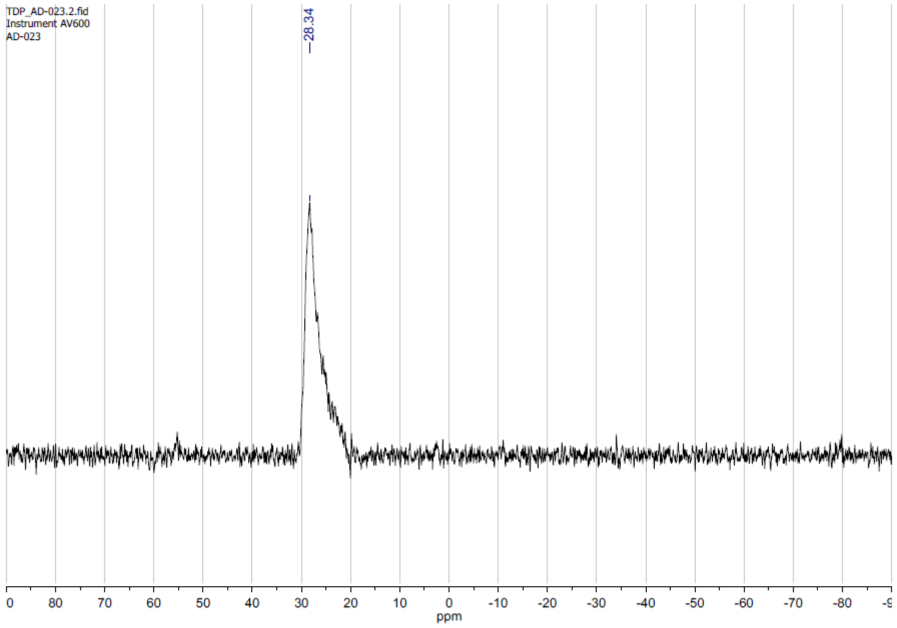

1-Hydroxythieno[3,2-*d*][1,2,3]diazaborinine-2(1*H*)-carboxamide (6)

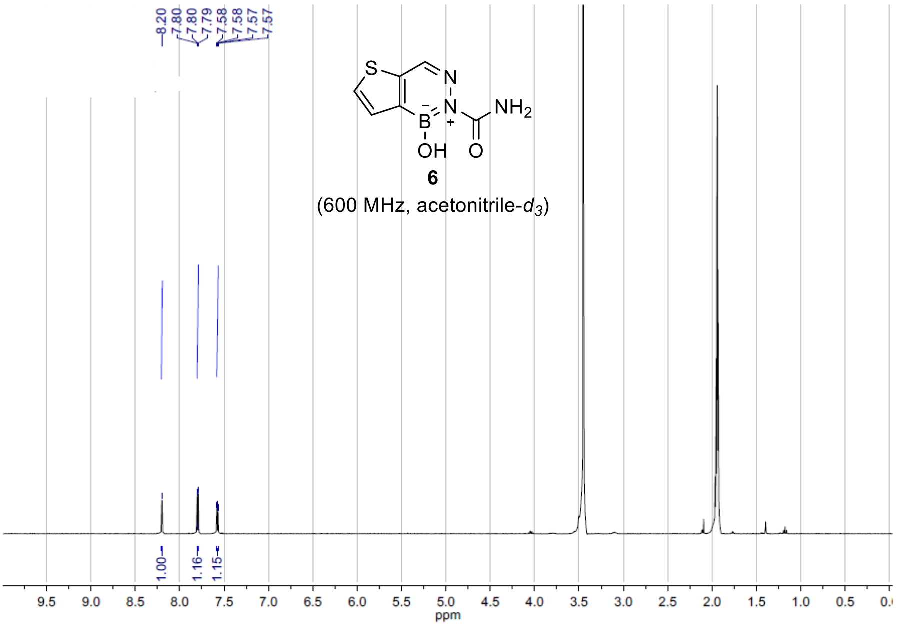

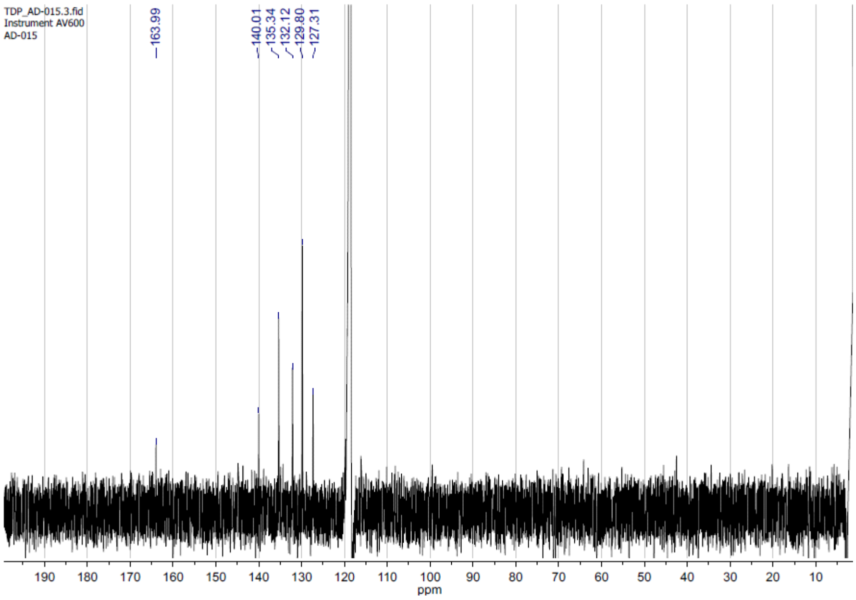

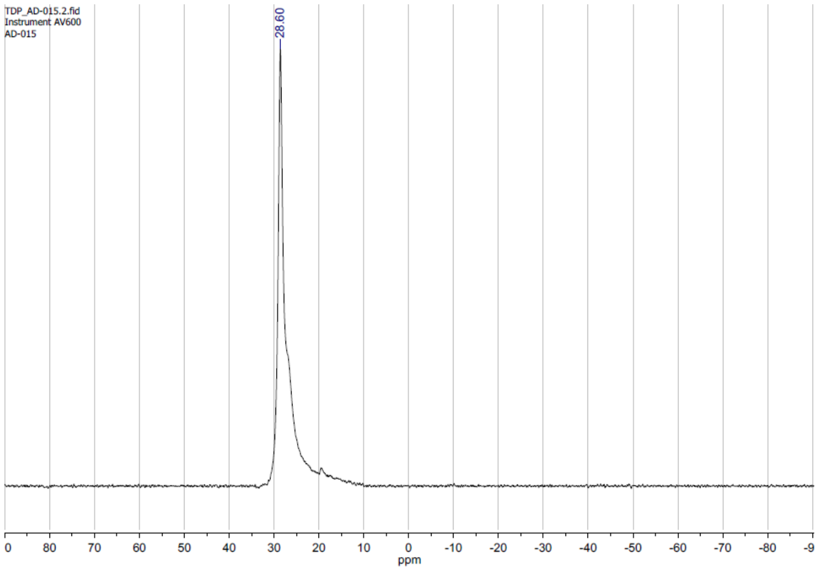

1-Hydroxy-4-methylthieno[3,2-*d*][1,2,3]diazaborinine-2(1*H*)-carboxamide (7)

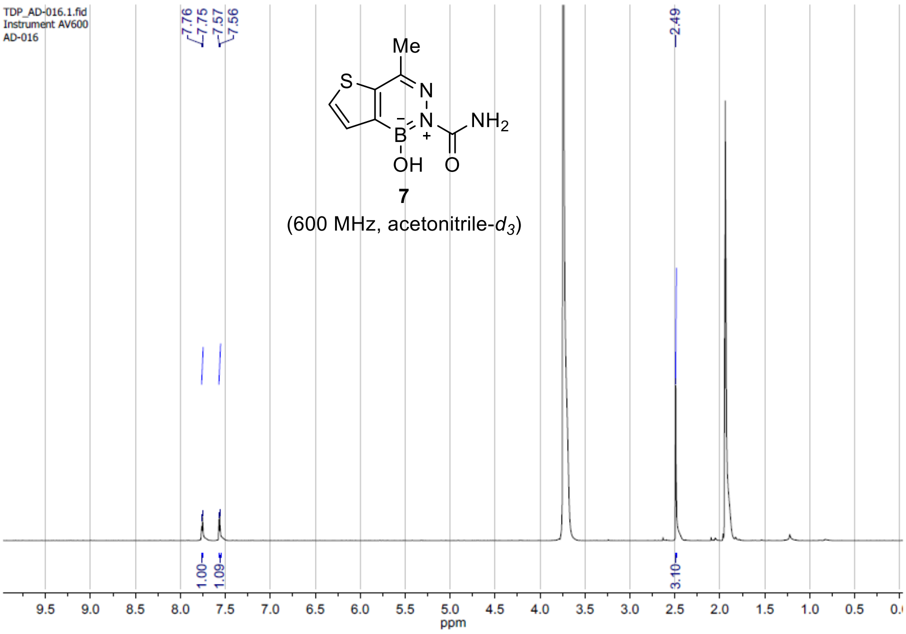

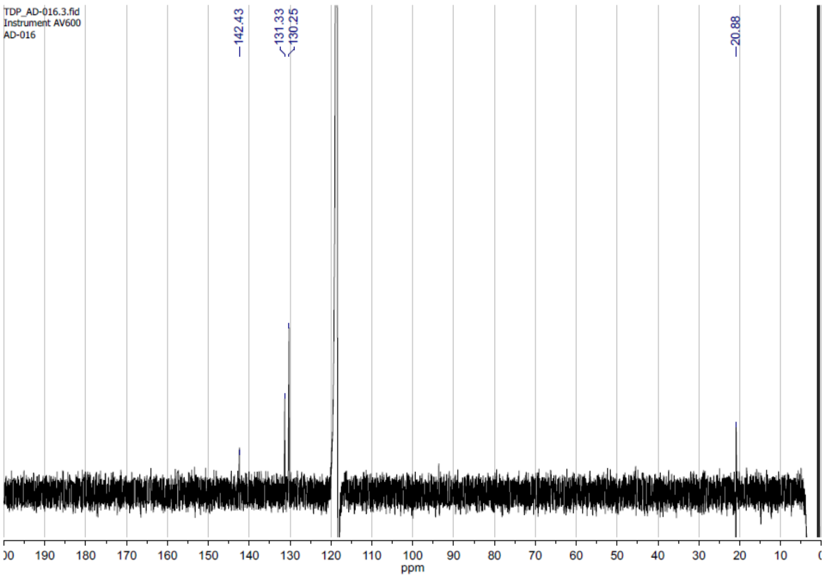

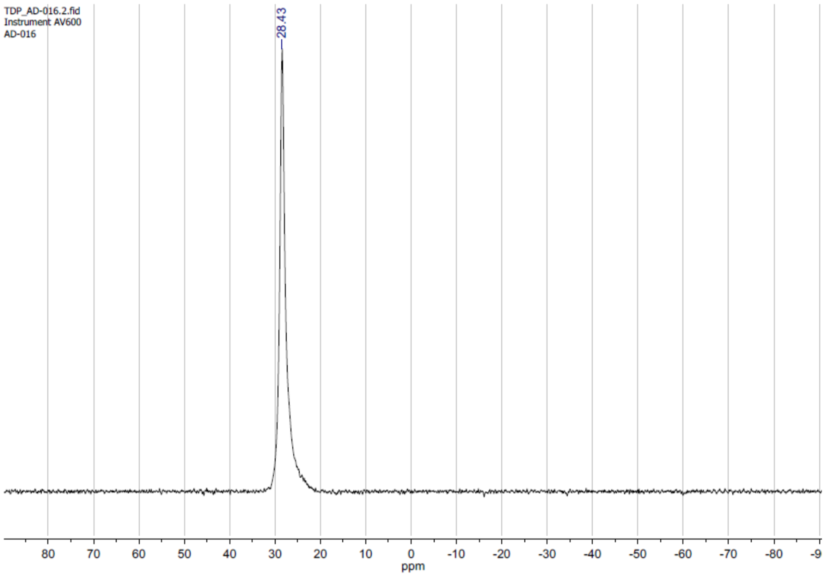

1-Hydroxythieno[2,3-*d*][1,2,3]diazaborinine-2(1*H*)-carboxamide (8)

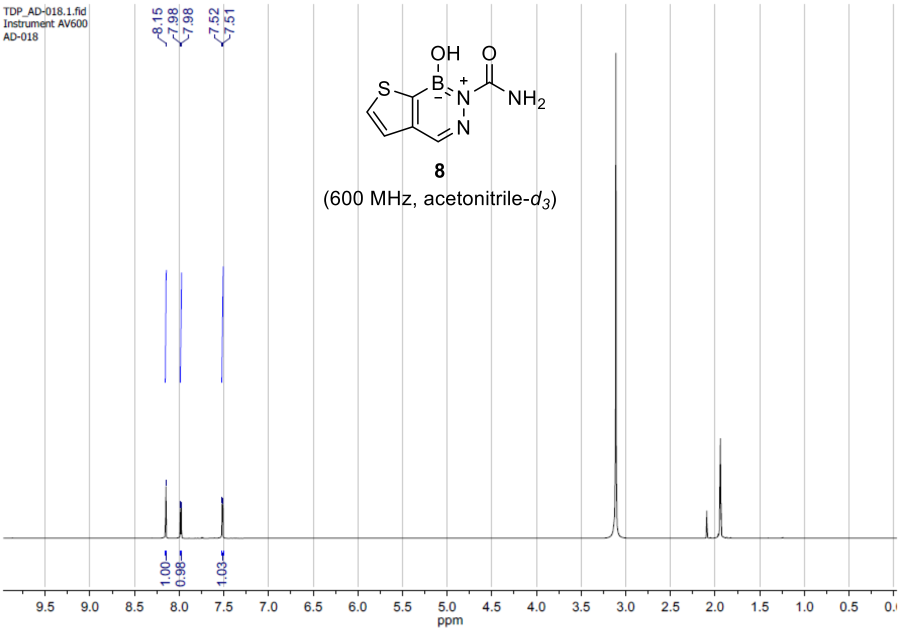

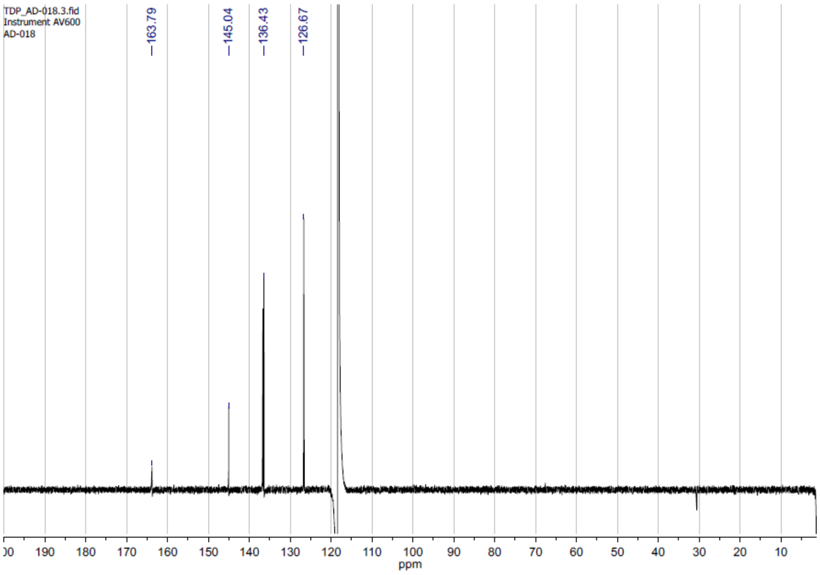

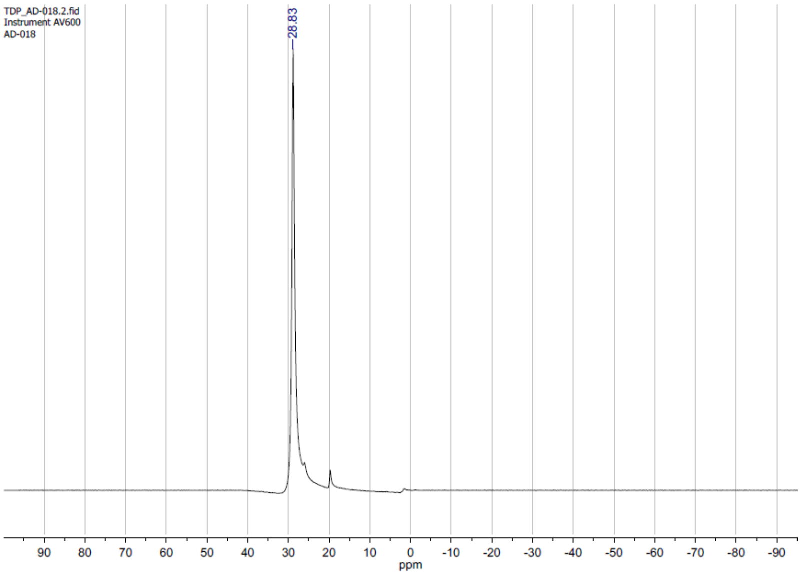

1-Hydroxybenzo[*d*][1,2,3]diazaborinine-2(1*H*)-carbothioamide (9)

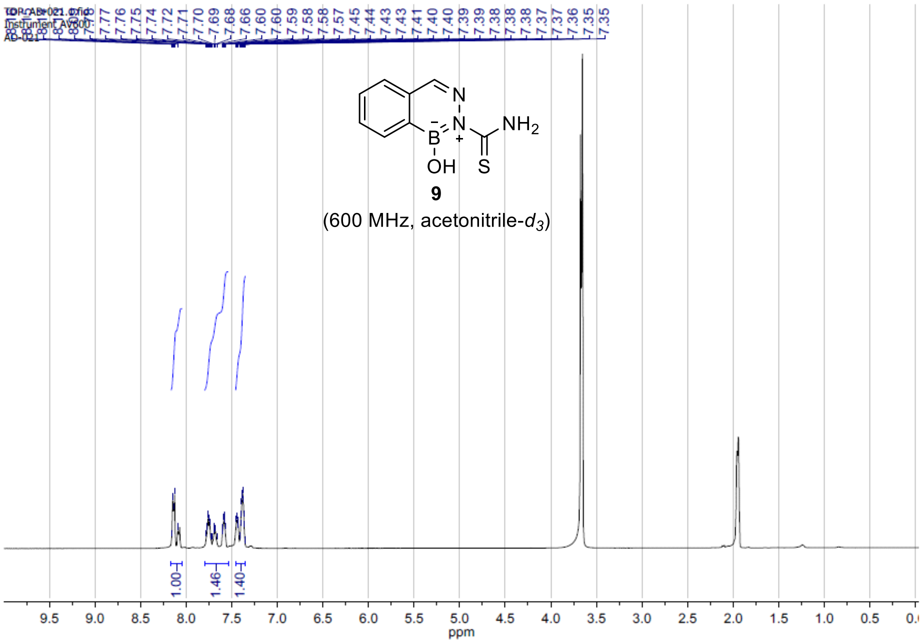

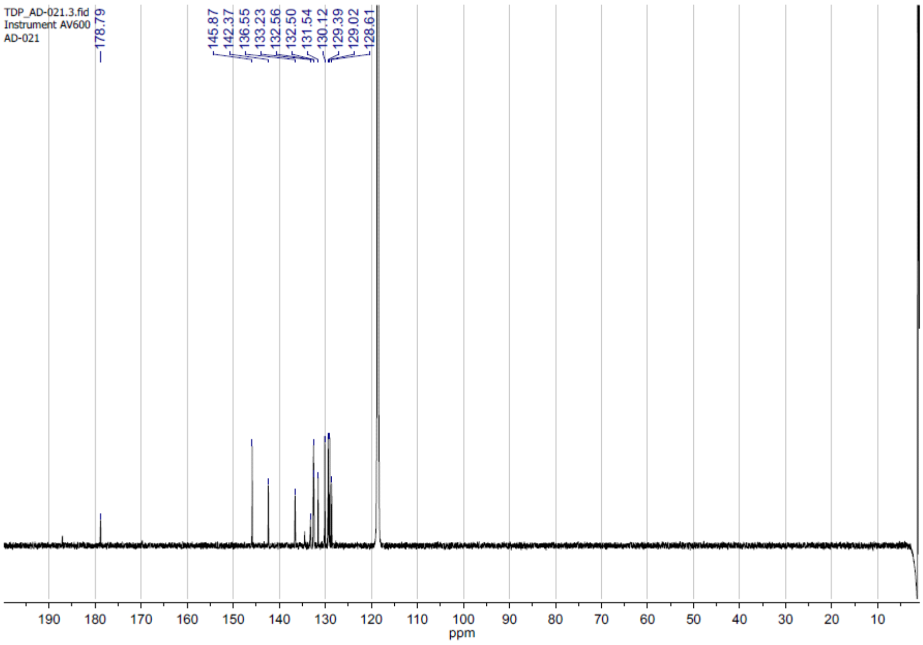

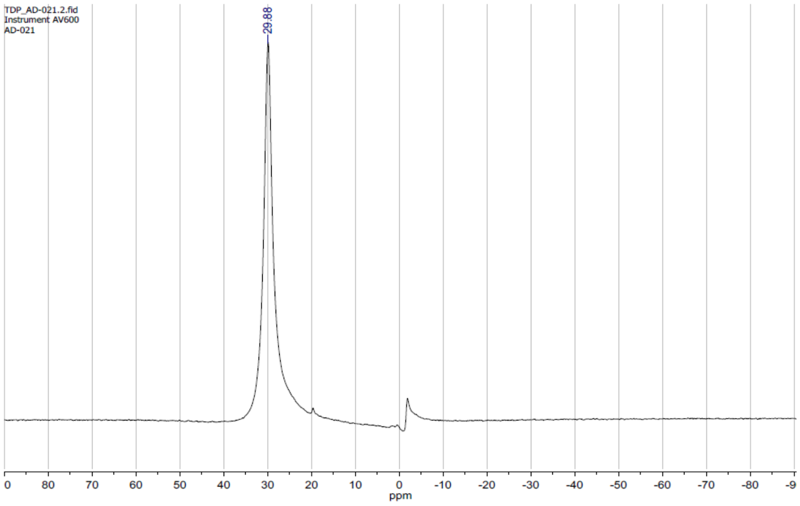

1-Hydroxy-4-methylbenzo[*d*][1,2,3]diazaborinine-2(1*H*)-carbothioamide (10)

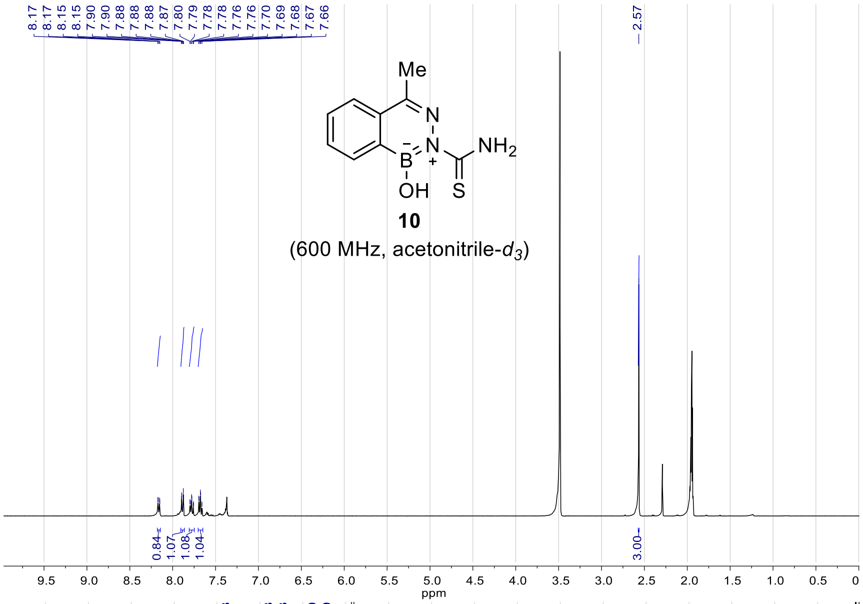

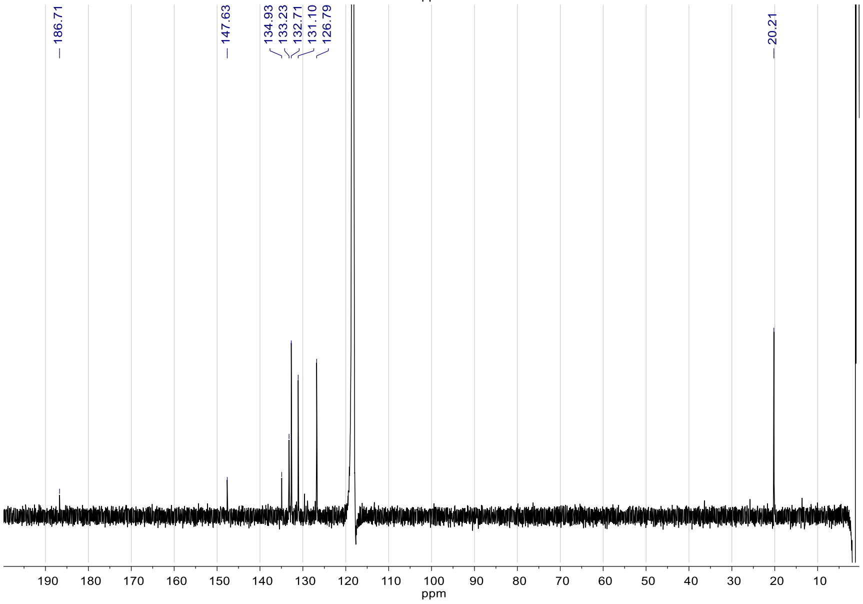

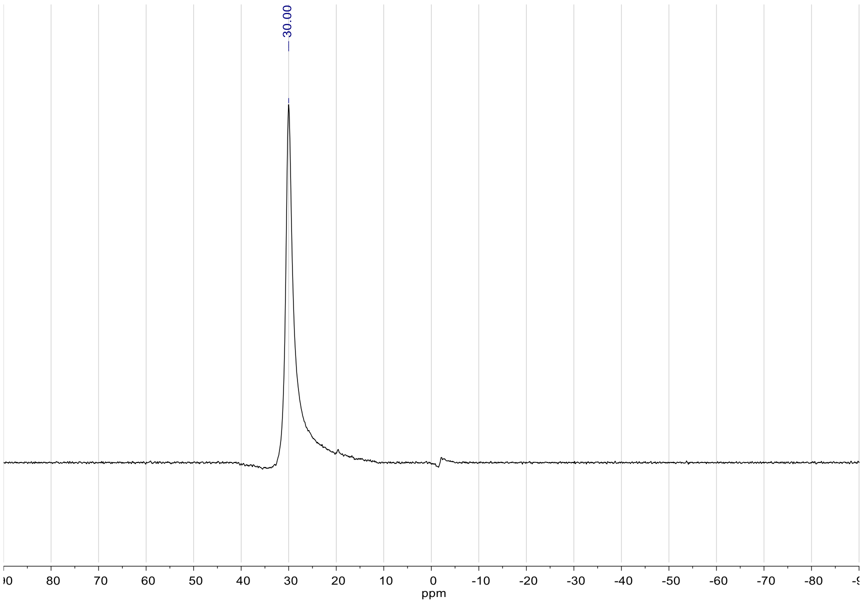

(2a*S*)-4a-Hydroxy-2,2a,4a,8b-tetrahydro-3*H*-4-oxa-1-thia-2a^{1}-aza-4aλ^{4}-borapentaleno[1,6-*ab*]inden-3-one (11)

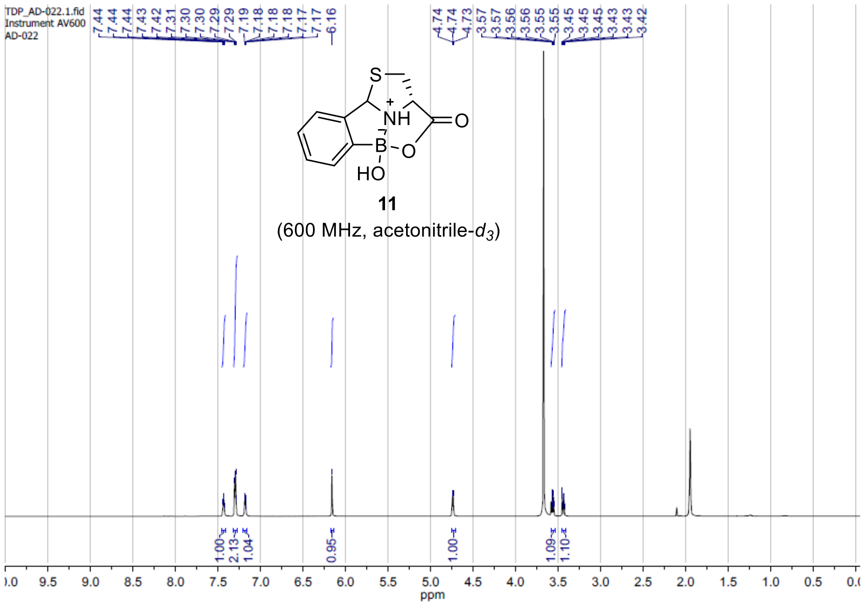

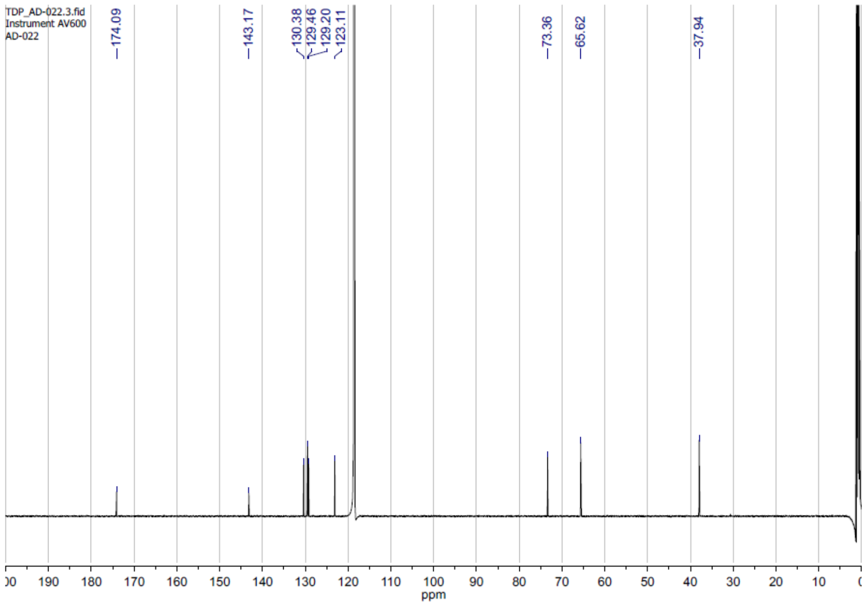

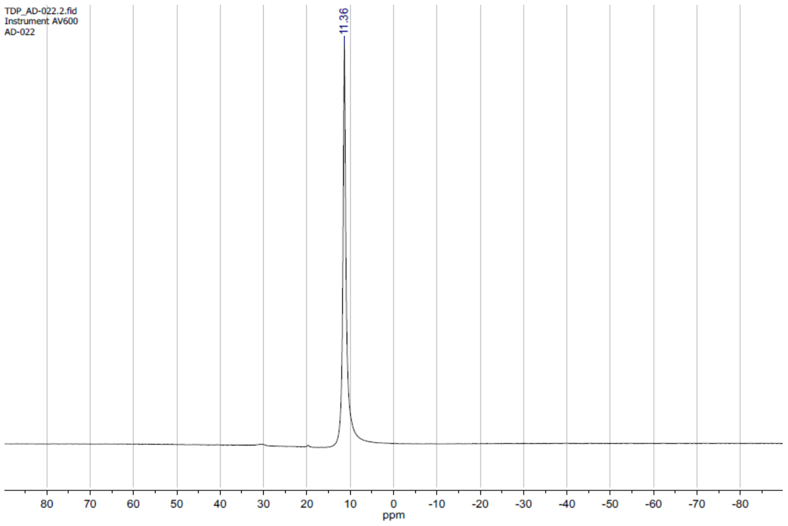

(±)-4a-Hydroxy-2,2-dimethyl-2,2a,4a,8b-tetrahydro-3*H*-4-oxa-1-thia-2a^{1}-aza-4aλ^{4}-borapentaleno[1,6-*ab*]inden-3-one (13) & (14)

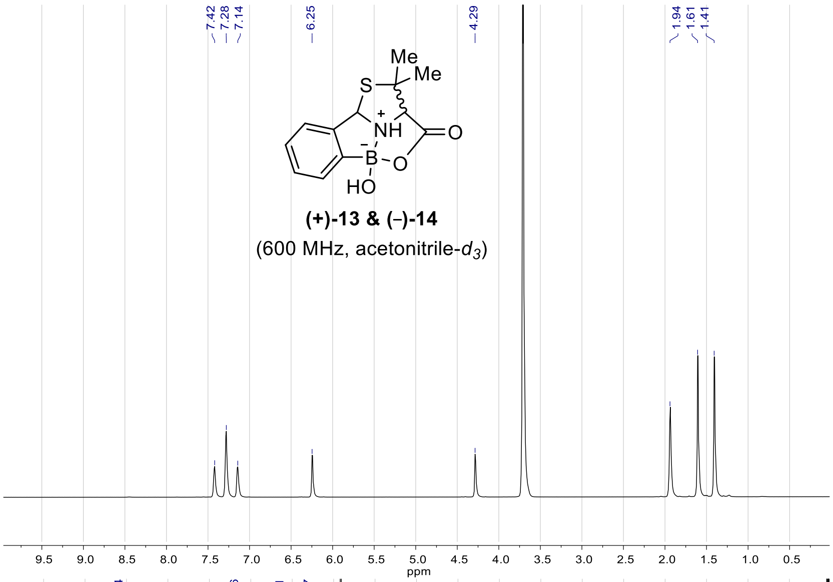

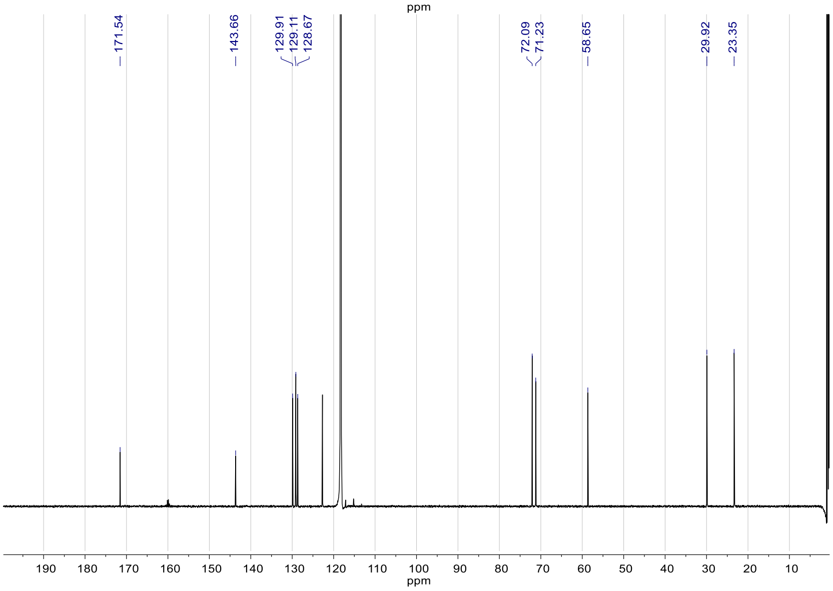

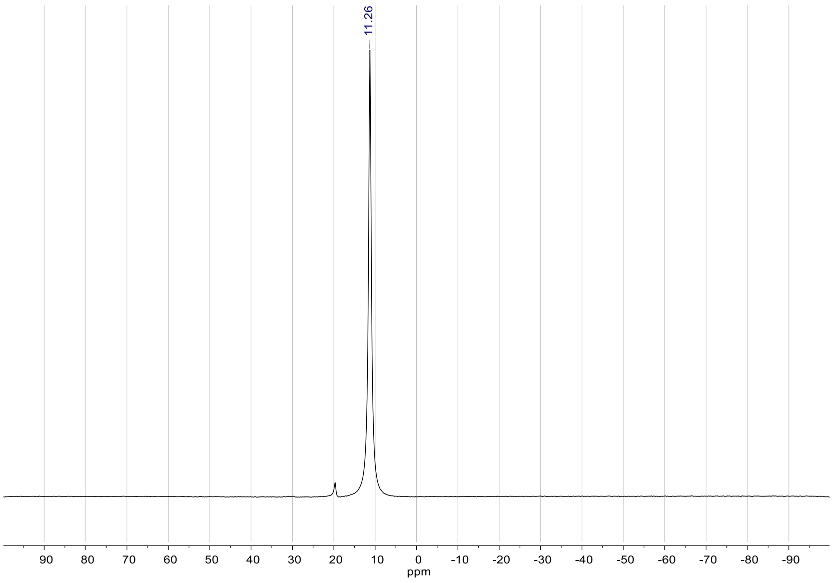

(2a*S*)-4a-Hydroxy-8b-methyl-2,2a,4a,8b-tetrahydro-3*H*-4-oxa-1-thia-2a^{1}-aza-4aλ^{4}-borapentaleno[1,6-*ab*]inden-3-one (15)

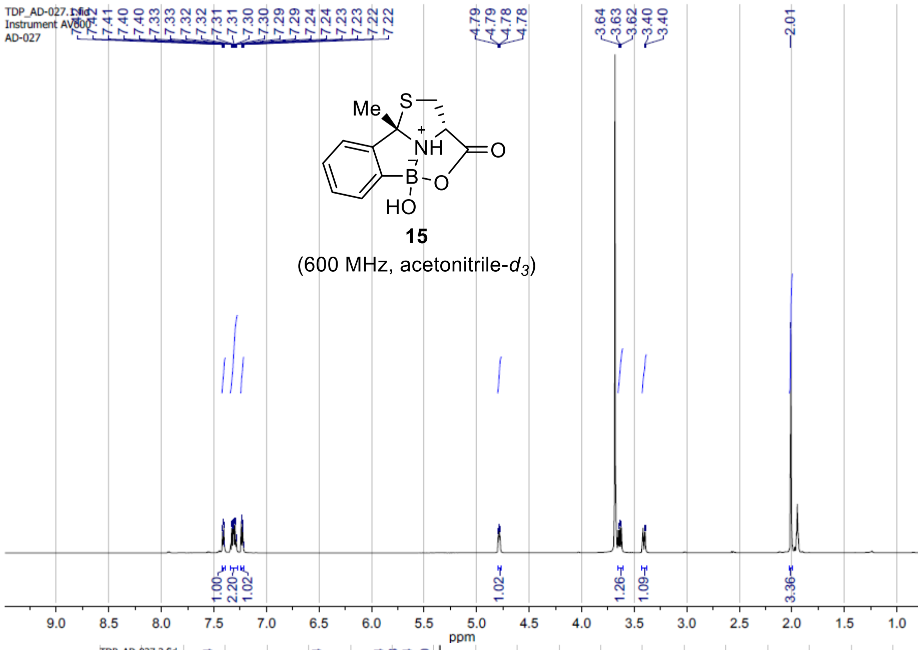

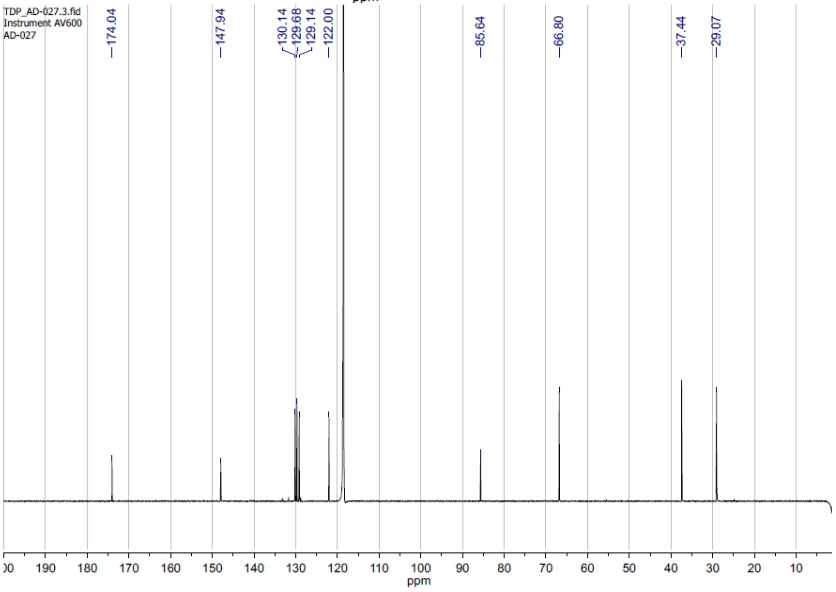

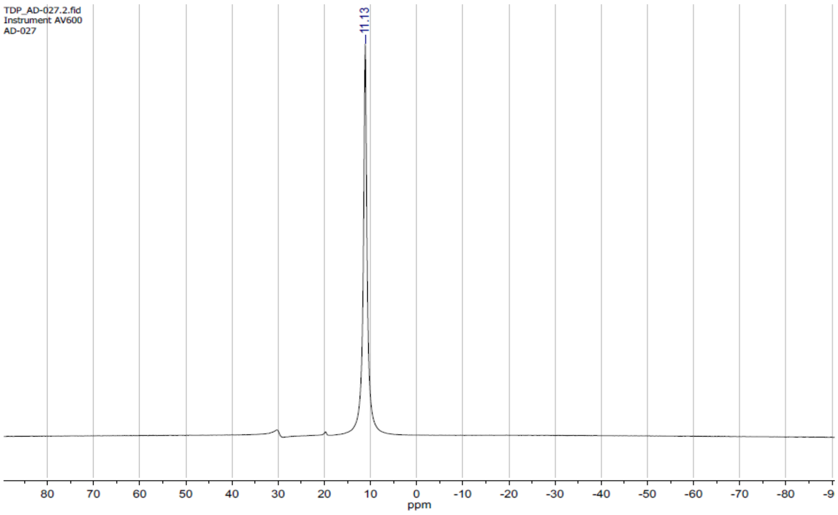

(2a*S*)-4a-Hydroxy-2,2,8b-trimethyl-2,2a,4a,8b-tetrahydro-3*H*-4-oxa-1-thia-2a^{1}-aza-4aλ^{4}-borapentaleno[1,6-*ab*]inden-3-one (17)

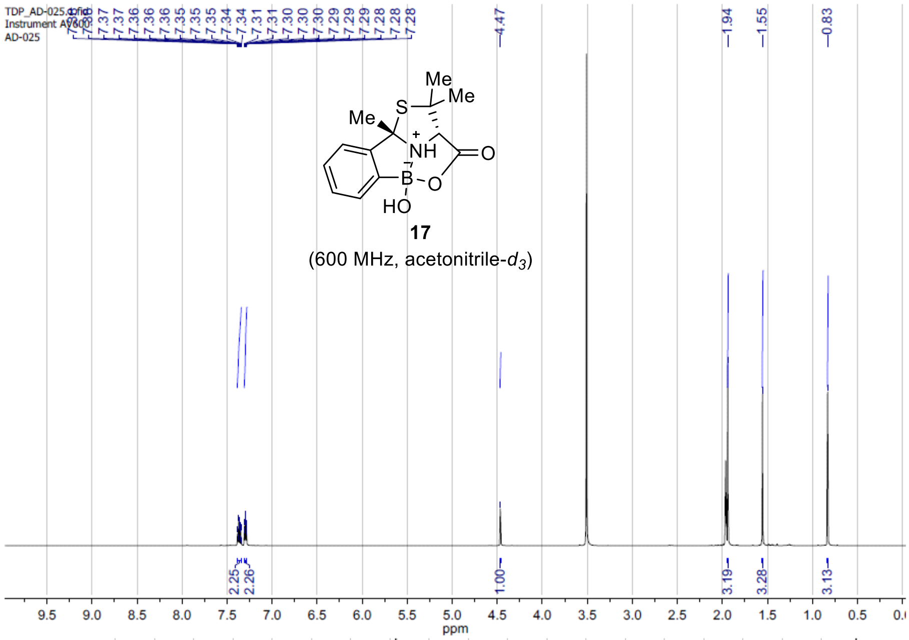

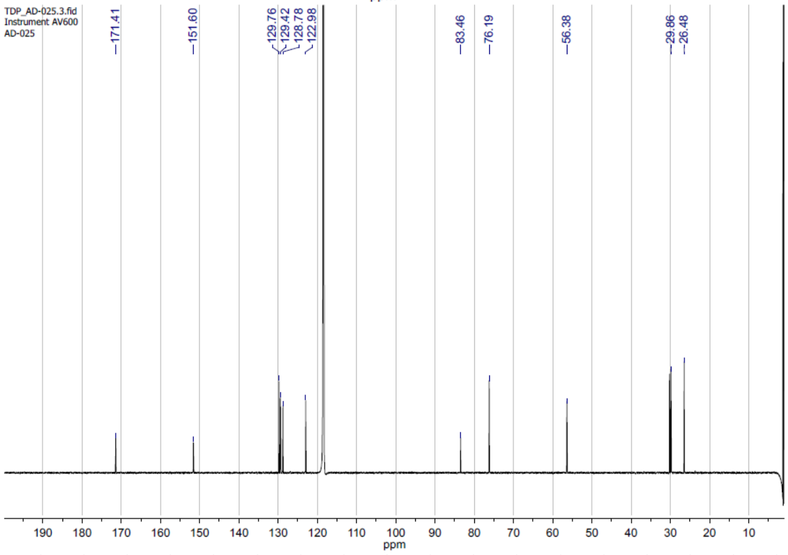

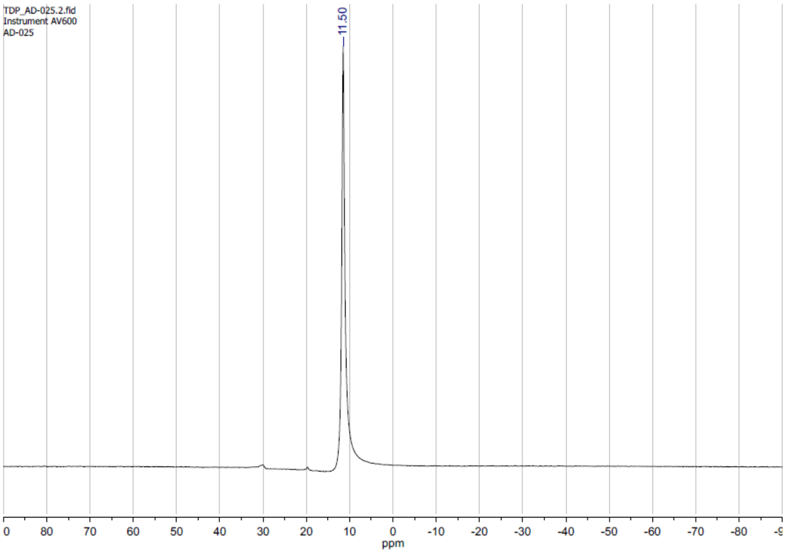

(*E*)-2-(Thiophen-3-ylmethylene)hydrazine-1-carboxamide (19)

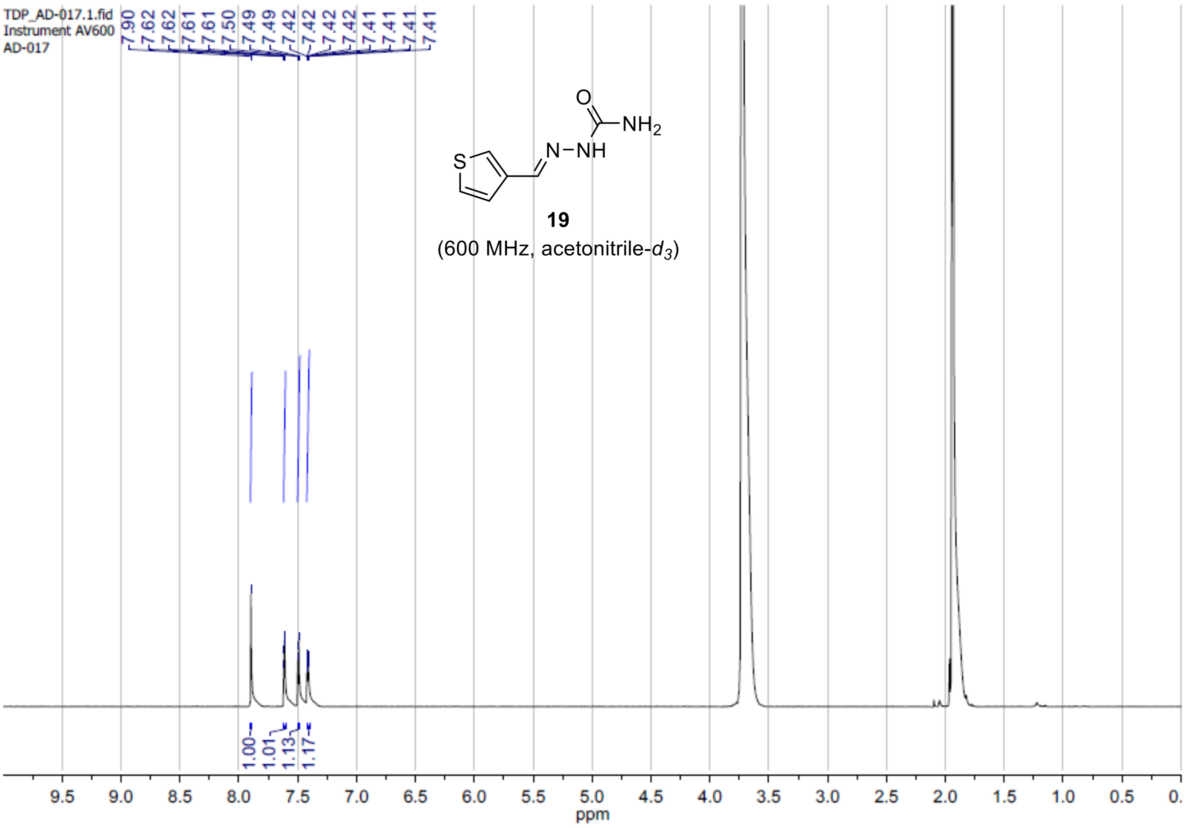

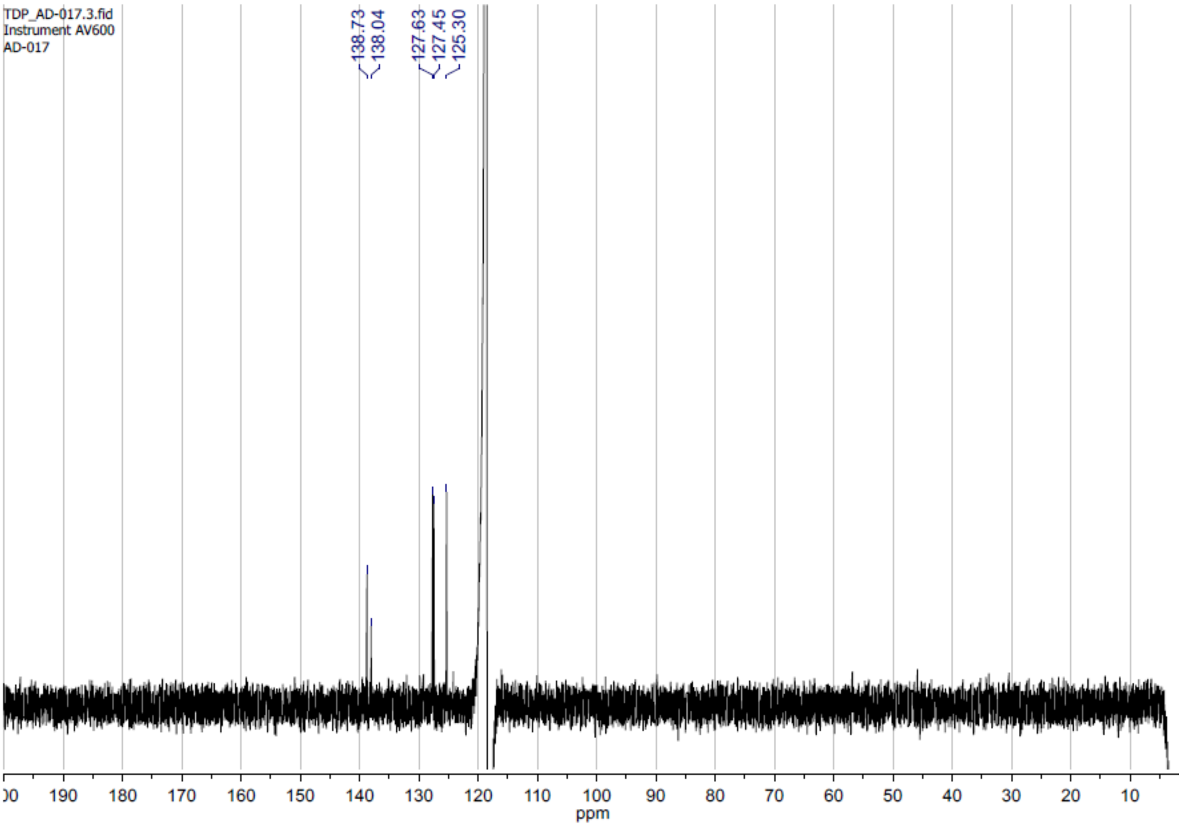

Propane-1-sulfonohydrazide (20)

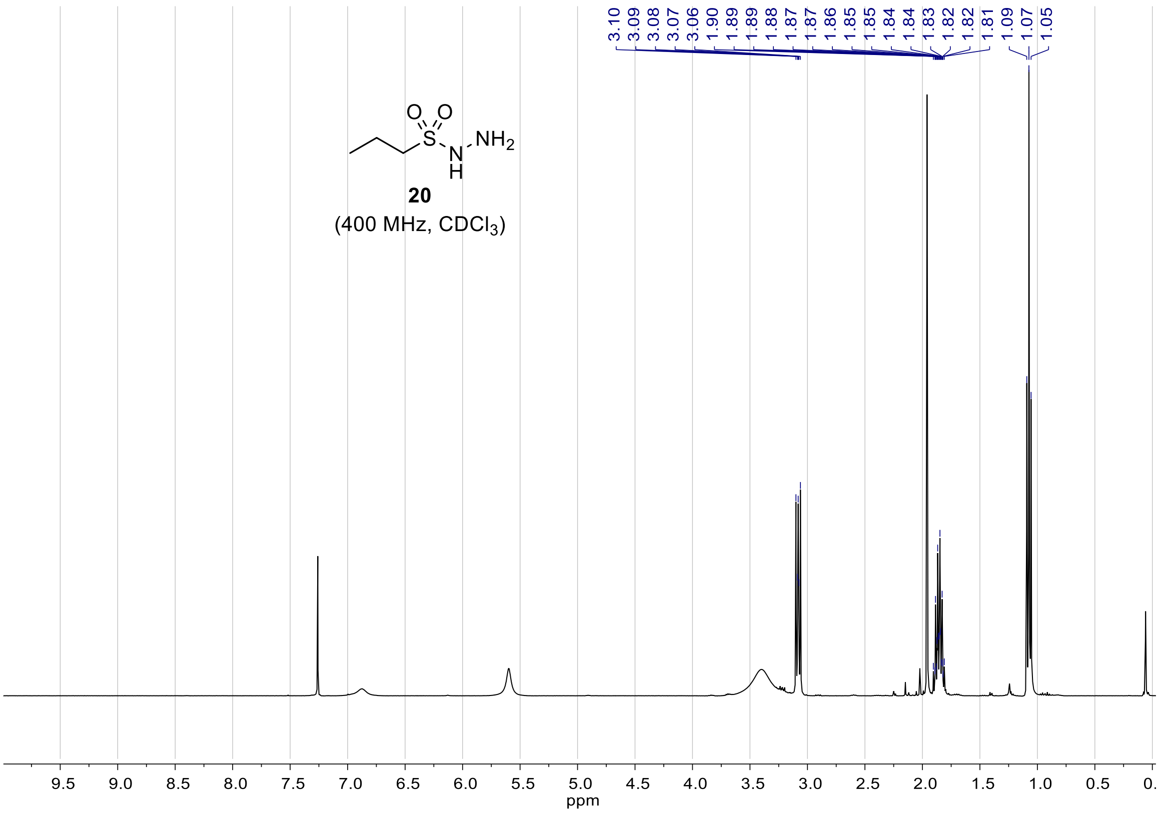

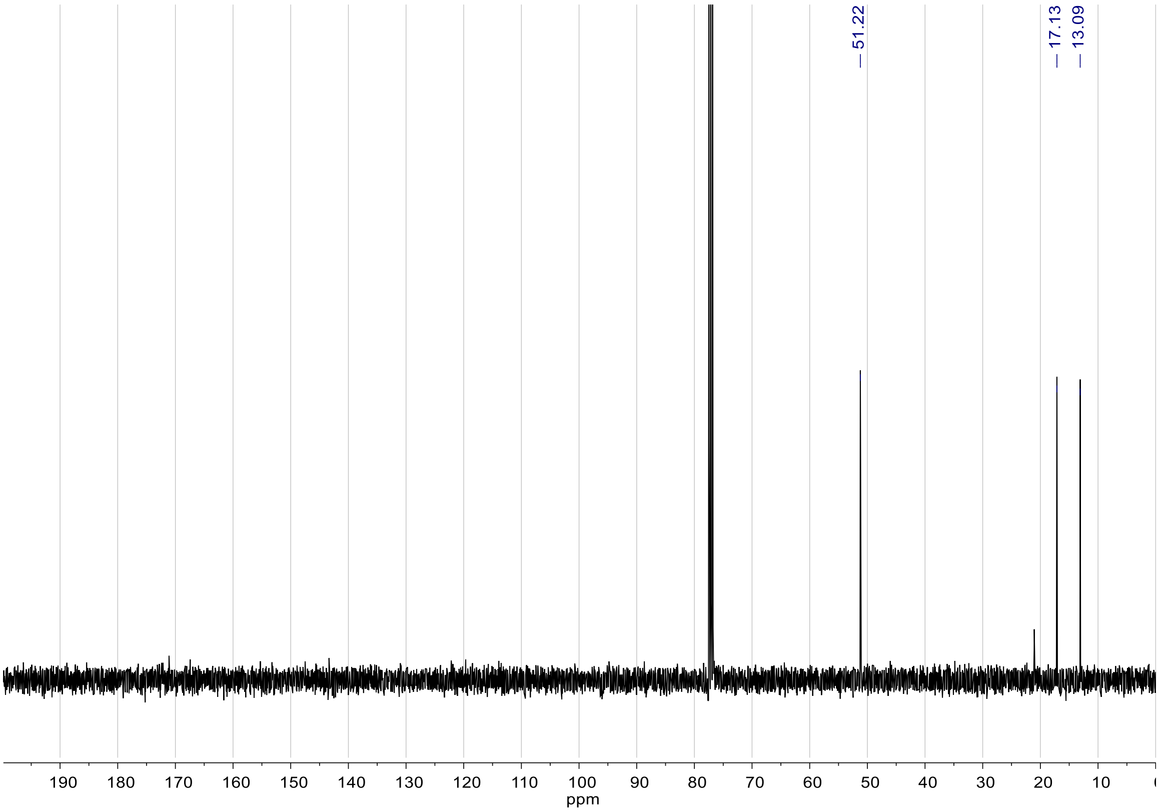

6-Methylthieno[3,2-*d*][1,2,3]diazaborinin-1(2*H*)-ol (21)

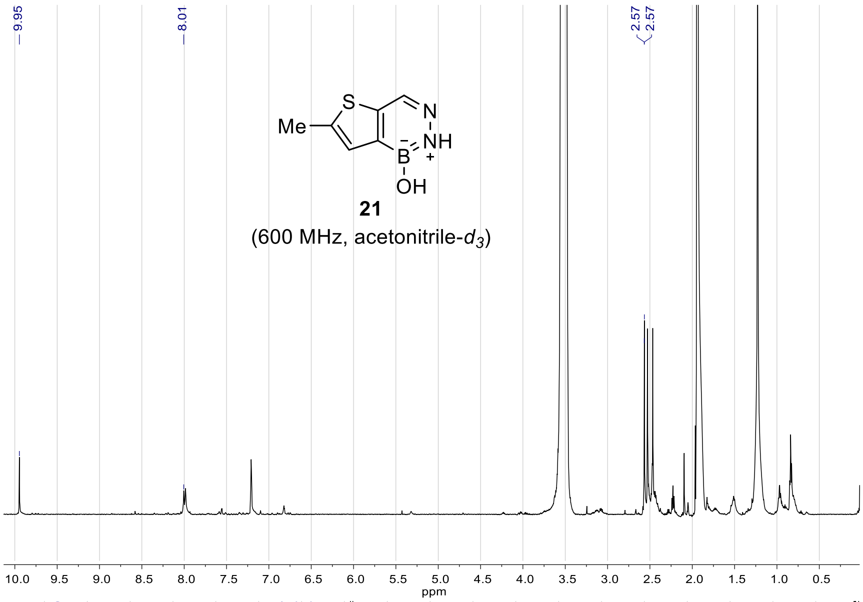

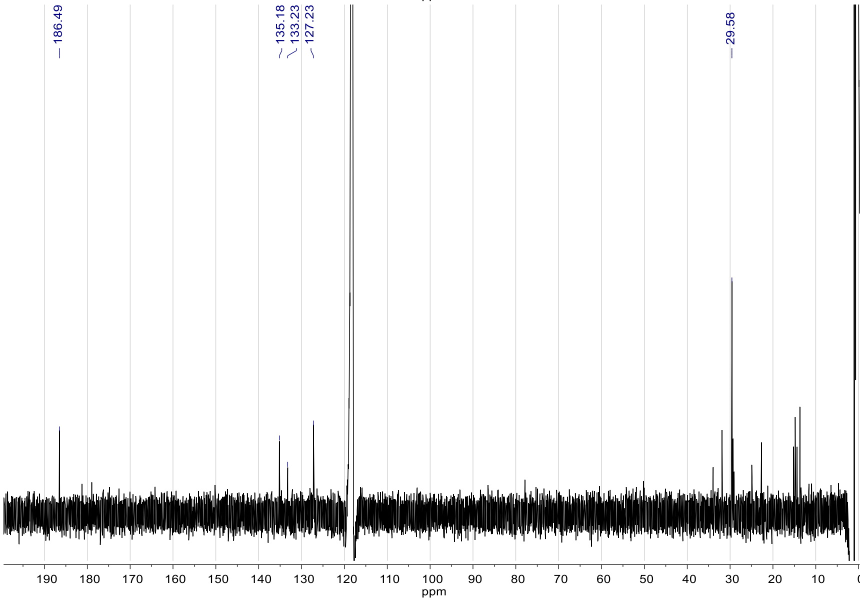

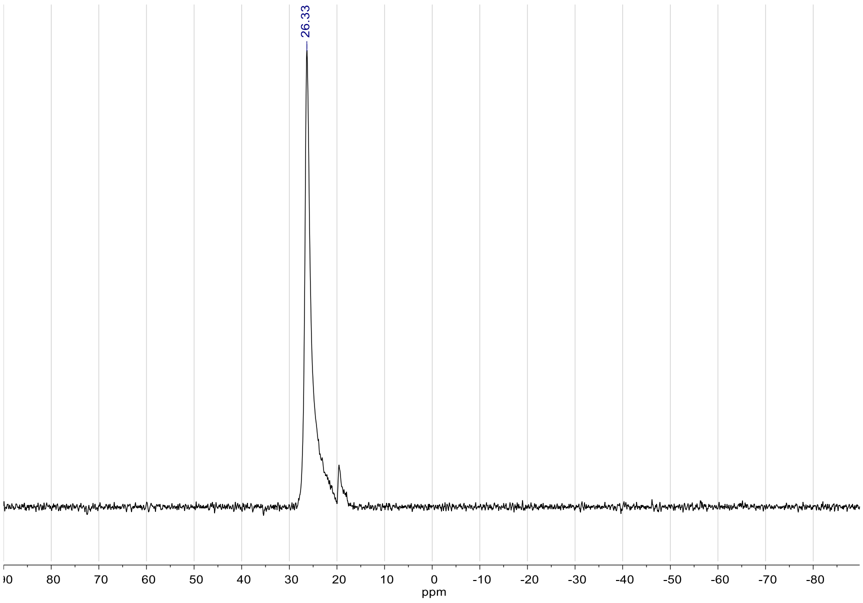

(*E*)-*N'*-((5-Methylthiophen-2-yl)methylene)propane-1-sulfonohydrazide (22)

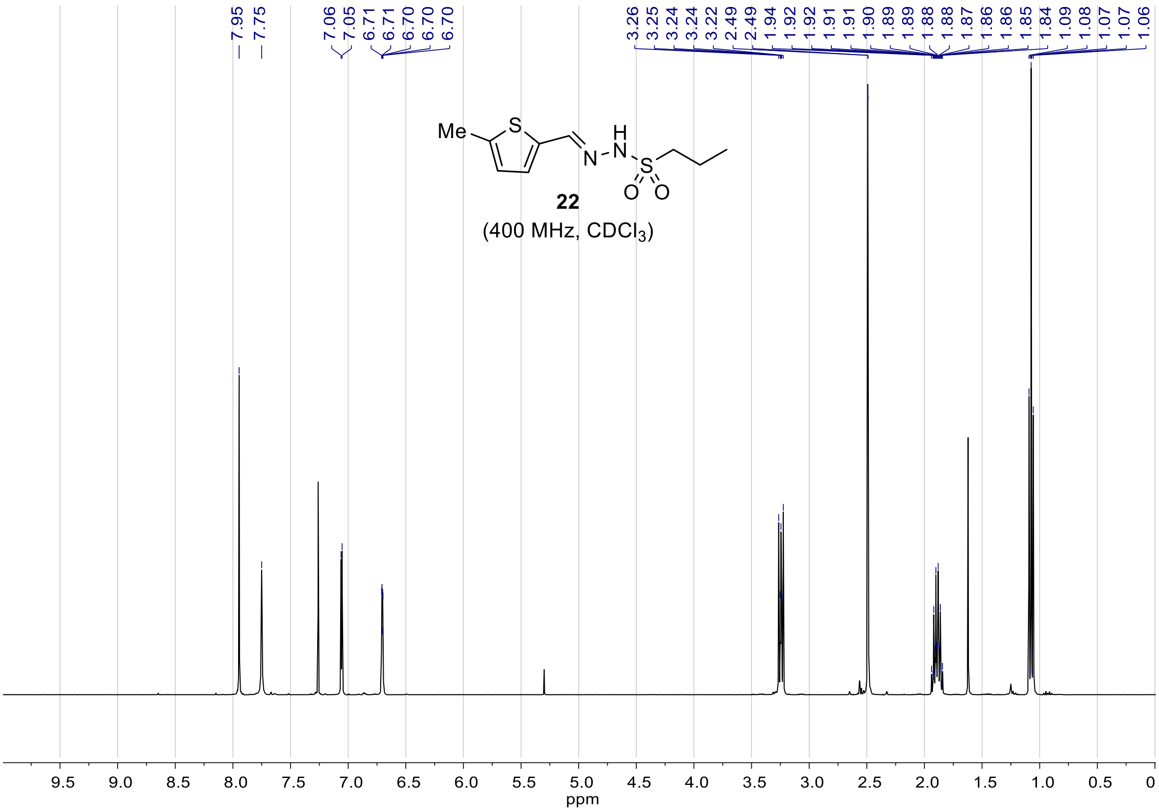

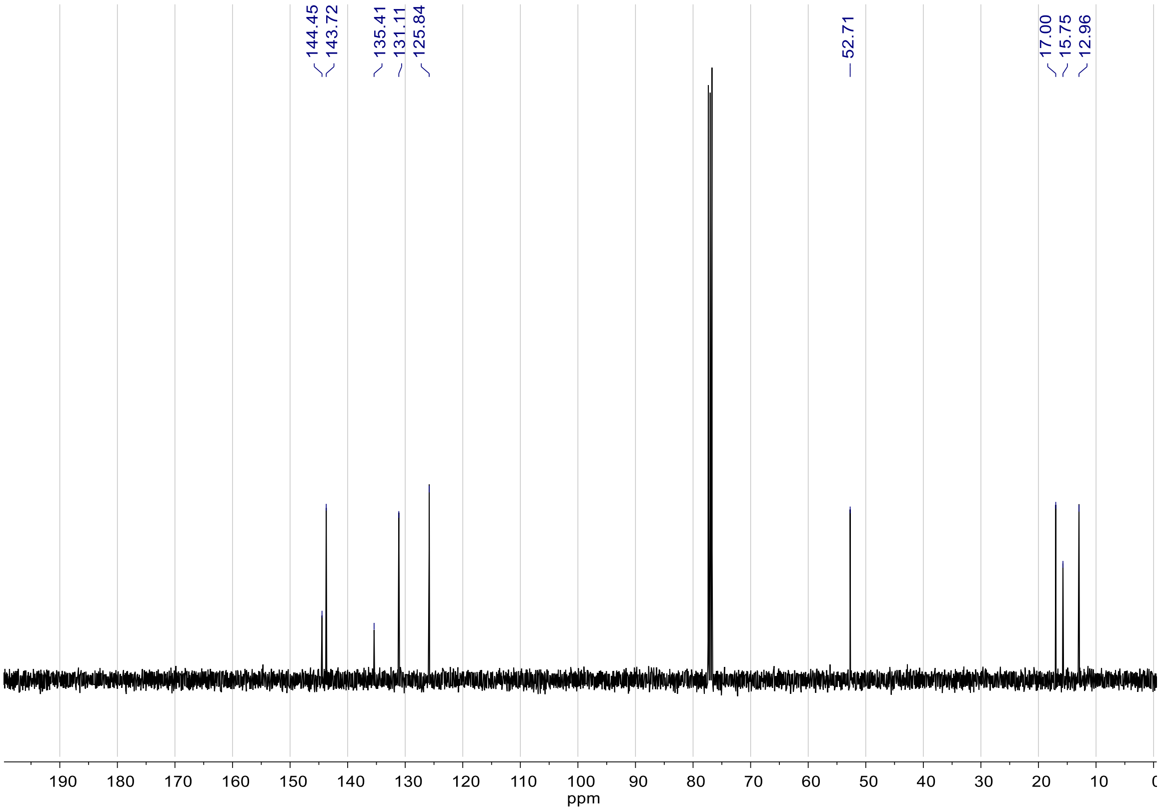

6-Methyl-2-(propylsulfonyl)thieno[3,2-*d*][1,2,3]diazaborinin-1(2*H*)-ol (23)

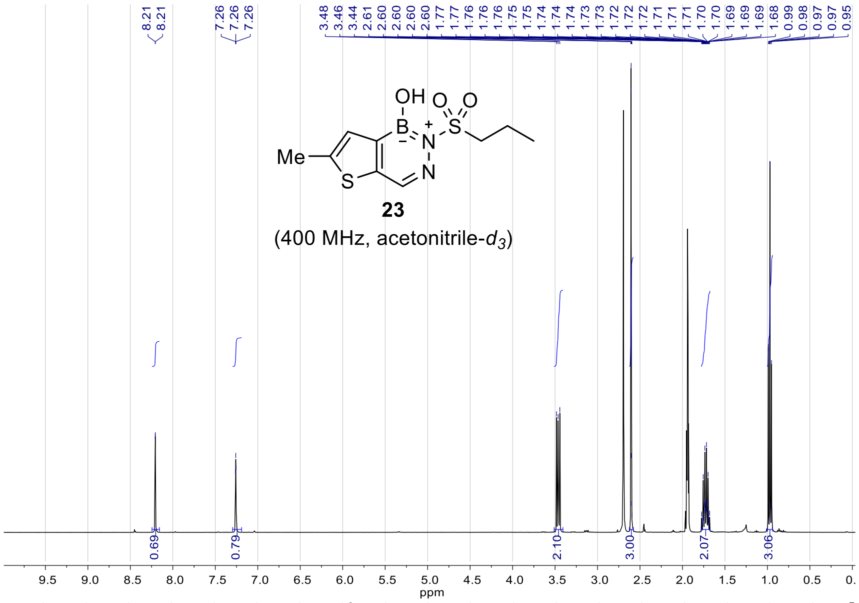

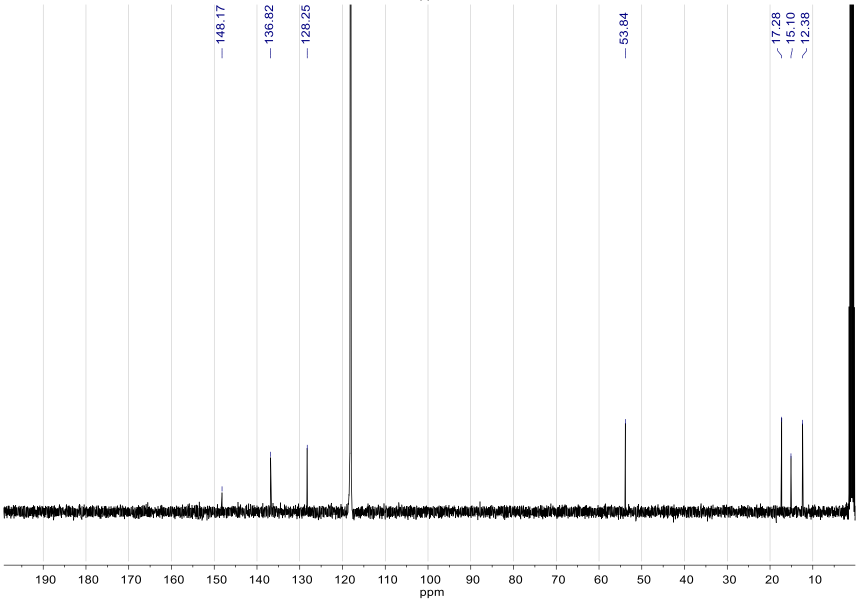

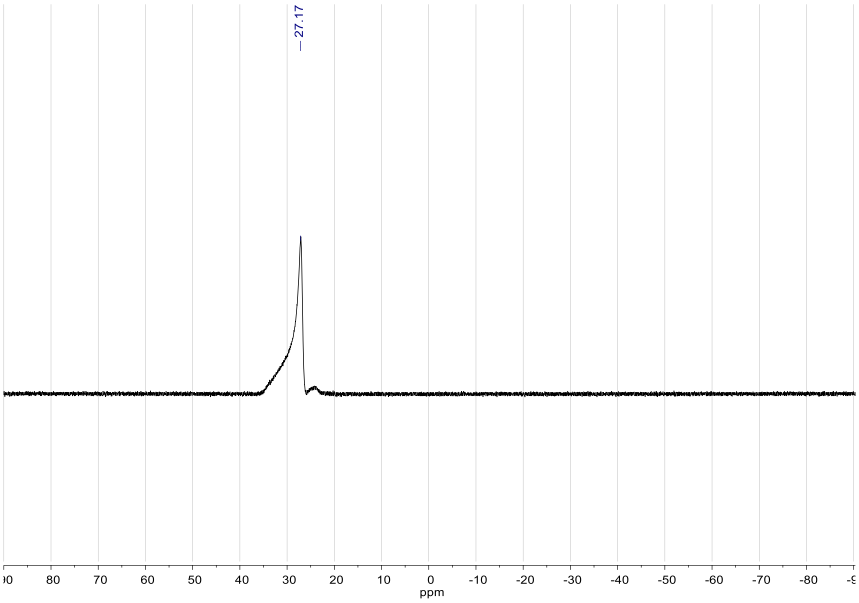

Supplement: Supplementary file 1 [file molecules-29-04998-s001.zip › molecules-3220618-supplementary.pdf]
